# ZDHHC2‐Dependent Palmitoylation Dictates Ferroptosis and Castration Sensitivity in Prostate Cancer via Controlling ACSL4 Degradation and Lipid Peroxidation

**DOI:** 10.1002/advs.202514077

**Published:** 2025-10-23

**Authors:** Shuai Shao, Wei Li, Yulong Hong, Ruijiang Zeng, Liang Zhu, Lu Yi, Yuan Li, Yinhuai Wang, Haojie Huang, Xuewen Jiang, Xin Jin

**Affiliations:** ^1^ Department of Urology The Second Xiangya Hospital Central South University Changsha Hunan 410011 China; ^2^ Key Laboratory of Diabetes Immunology (Central South University), Ministry of Education National Clinical Research Center for Metabolic Disease Changsha 410011 China; ^3^ Uro‐Oncology Institute of Central South University Changsha Hunan 410011 China; ^4^ Furong Laboratory Changsha Hunan 410000 China; ^5^ Institute of Urologic Science and Technology The First Affiliated Hospital Zhejiang University School of Medicine Hangzhou 310003 China; ^6^ Department of Urology Qilu Hospital Cheeloo College of Medicine Jinan 250200 China; ^7^ Key Laboratory of Urinary Precision Diagnosis and Treatment Universities of Shandong Jinan 250200 China; ^8^ Shandong Provincial Engineering Research Center for Precision Diagnosis and Treatment of Urological and Male Reproductive Diseases Jinan 250200 China

**Keywords:** ACSL4, CRPC, ferroptosis, lipid peroxide production, ZDHHC2

## Abstract

Ferroptosis represents a promising vulnerability to overcome therapeutic resistance in castration‐resistant prostate cancer (CRPC). While S‐palmitoylation of lipid peroxide‐scavenging proteins such as GPX4 and SLC7A11 has been shown to suppress ferroptosis, whether palmitoylation modulates the lipid peroxidation generation remains unclear. Here, we identified the palmitoyltransferase ZDHHC2 as a critical driver of enzalutamide resistance through destabilizing ACSL4. ZDHHC2 is transcriptionally upregulated by a FOXA1/CXXC5/TET2 complex and promotes S‐palmitoylation of the deubiquitinase USP19, which impairs its interaction with ACSL4. This disrupts USP19‐mediated ACSL4 stabilization, promoting its ubiquitin–proteasome degradation and consequently suppressing lipid peroxidation and ferroptosis. We synthesized a small‐molecule ZDHHC2 inhibitor, TTZ1, which restores ACSL4 protein, reactivates ferroptosis, and reverses enzalutamide resistance in CRPC cell lines and patient‐derived xenograft models. This study uncovers a previously unrecognized mechanism by which palmitoylation regulates ferroptosis through modulating ACSL4 stability, and highlights the ZDHHC2‐USP19‐ACSL4 axis as a druggable target for overcoming resistance in advanced prostate cancer.

## Graphical abstract

Our study reveals a novel mechanism by which palmitoylation regulates ferroptosis and enzalutamide resistance in prostate cancer. Under enzalutamide resistance, the FOXA1/CXXC5/TET2 complex induces aberrant upregulation of ZDHHC2, which subsequently suppresses ferroptosis by mediating S‐palmitoylation of USP19 and degradation of ACSL4 protein in CRPC cells. We synthesized TTZ1, a specific inhibitor of ZDHHC2 that can intervene in this process.

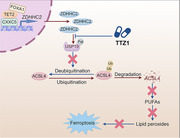

## 1 Introduction

The reactivation of the androgen receptor (AR) signaling pathway is considered the major driver of castration‐resistant prostate cancer (CRPC).^[^ ^1^ ^]^ To inhibit AR signaling in CRPC, next‐generation AR signaling inhibitors such as enzalutamide were developed.^[^ ^2^ ^]^ However, with their widespread use, resistance to next‐generation AR signaling inhibitors has emerged as a major clinical problem.^[^ ^2^, ^3^, ^4^, ^5^ ^]^ Lipid biosynthesis and metabolism‐related biological processes regulate prostate cancer progression and enzalutamide resistance.^[^ ^6^, ^7^, ^8^, ^9^ ^]^ Sphingolipids‐a specific class of phospholipids encompassing sphingomyelins, ceramides, and sphingosine‐1‐phosphate (S1P)—actively regulate cell signaling, proliferation, survival, and migration.^[^ ^10^, ^11^, ^12^ ^]^ Crucially, ceramide and S1P exhibit anti‐tumor and pro‐tumor effects, respectively, with S1P concentrations elevated in multiple cancer types.^[^ ^13^ ^]^ Recent studies indicate that resistance to AR signaling inhibitors arises not only from AR genetic alterations but also from perturbations in sphingolipid metabolism. Consequently, combining AR signaling inhibition with sphingolipid metabolism modulation could potentially benefit men with CRPC.^[^ ^14^ ^]^ Palmitic acid and palmitoyl‐CoA, which serve as the primary substrates for sphingolipid synthesis, were found to be involved not only in energy metabolism but also in protein S‐palmitoylation.^[^ ^15^, ^16^ ^]^ S‐palmitoylation, a reversible post‐translational lipid modification catalyzed by zinc finger aspartate–histidine–histidine–cysteine (DHHC)‐type palmitoyltransferase has been identified as a regulatory key in cancers by altering location, trafficking, stability, protein‐protein interactions, and function of proteins.^[^ ^17^ ^]^ However, the impact of protein palmitoylation modification mechanisms on enzalutamide sensitivity remains unknown.

Ferroptosis is an iron‐dependent form of cell death driven by phospholipid peroxidation, playing a critical role in cancer progression and treatment resistance.^[^ ^18^ ^]^ The accumulation level of lipid peroxidation products, regulated by the balance between their production and elimination, is recognized as a determinant of ferroptosis initiation.^[^ ^19^, ^20^ ^]^ Key scavenging enzymes that suppress ferroptosis include ferroptosis suppressor protein 1, glutathione peroxidase 4 (GPX4), and GTP cyclohydrolase‐1.^[^ ^21^, ^22^ ^]^ Recent studies indicate that S‐palmitoylation serves as a key regulator of lipid peroxidation clearance pathways. ZDHHC20‐mediated palmitoylation of GPX4 at Cys66 site enhances GPX4 protein stability and promotes cancer progression by inhibiting ferroptosis, which can be reversed by APT2. And the general palmitoylation inhibitor 2‐BP has been shown to promote ferroptosis.^[^ ^23^ ^]^ Another research identified ZDHHC8‐mediated palmitoylation of GPX4 at Cys75 site, which suppresses the CD8+ cytotoxic T cell‐induced tumor cell ferroptosis, resulting in immune escape. And this process can be reversed by PF‐670462, the specific inhibitor of ZDHHC8 inhibitor.^[^ ^24^ ^]^ Palmitoylation of SLC7A11, another key ferroptosis regulator, has also been reported. ZDHHC8‐mediated palmitoylation of SLC7A11 at Cys327 site reduces its ubiquitination level, prevents lysosome‐mediated degradation, and inhibits ferroptosis.^[^ ^25^ ^]^ However, there is limited exploration on the relationship of S‐palmitoylation in lipid peroxide production regulators that promote ferroptosis. Notably, while current research has primarily focused on the SLC7A11/GPX4‐mediated lipid peroxide elimination pathway, it remains unknown whether palmitoylation modifications could also regulate the lipid peroxidation generation pathway, ultimately affecting ferroptosis and enzalutamide resistance.

In this study, our multi‐omics analyses revealed the critical role of palmitoylation modifications and ZDHHC2 in enzalutamide resistance. From a biological perspective, we discovered a novel regulatory function of palmitoylation in modulating the ferroptosis pathway. Mechanistically, enzalutamide activates a FOXA1‐dependent pathway to upregulate ZDHHC2, a palmitoyltransferase that drives S‐palmitoylation of ubiquitin carboxyl‐terminal hydrolase 19 (USP19) and promotes ACSL4 ubiquitination degradation. This effect inhibits lipid peroxidation and ferroptosis, conferring therapeutic resistance. Moreover, we synthesized the specific enzymatic inhibitor of ZDHHC2‐TTZ1,^[^ ^26^ ^]^ and showed that TTZ1 restores ACSL4 levels, reinstates ferroptosis sensitivity, and overcomes enzalutamide resistance in prostate cancer.

## 2 Results

### 2.1 ZDHHC2 Contributes to the Survival of CRPC Cells After Enzalutamide Treatment

**Figure 1:**
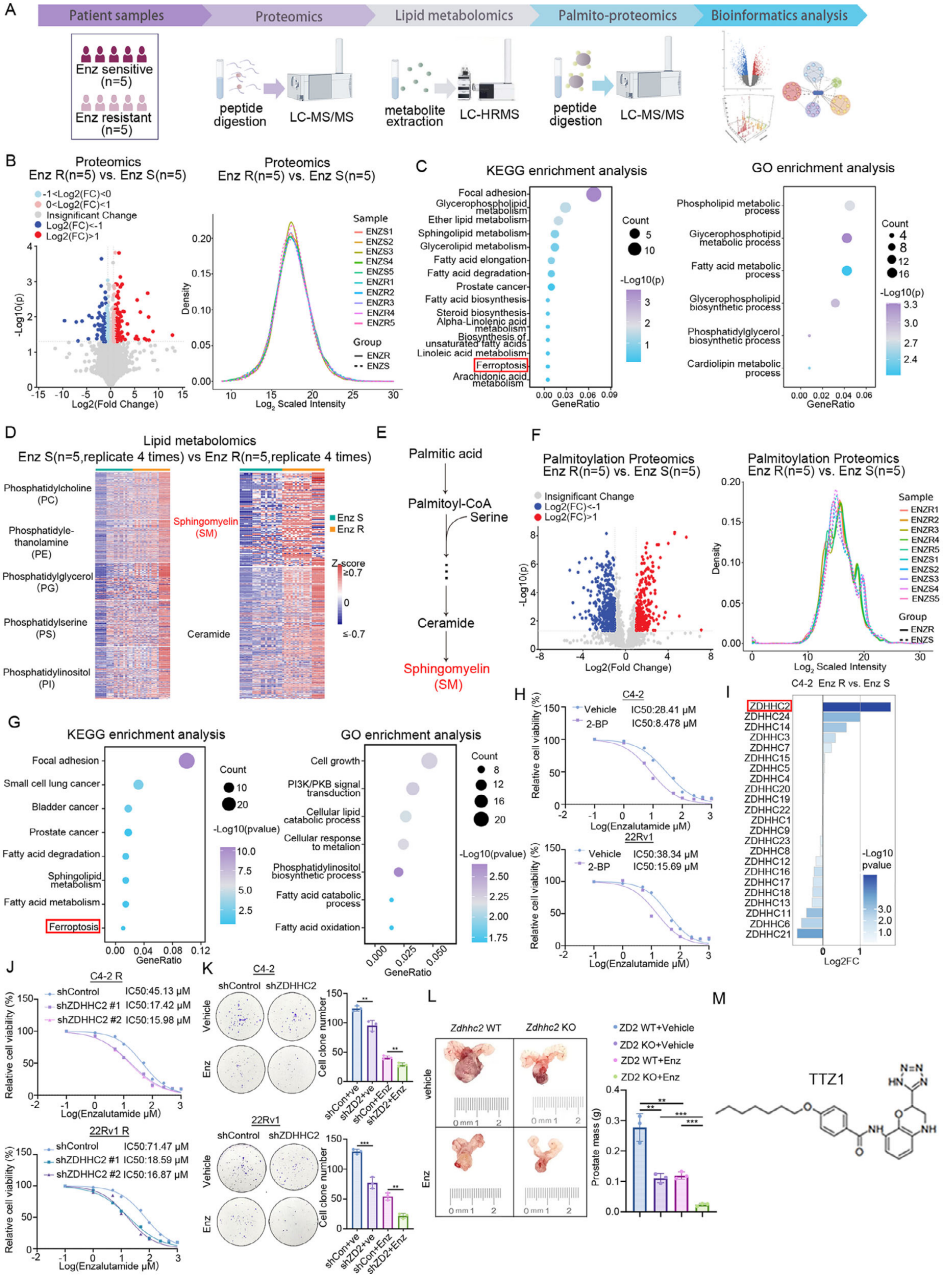
ZDHHC2 contributes to the survival of CRPC cells after enzalutamide treatment. A) flowchart illustrating the collection of enzalutamide‐sensitive (*n* = 5) and ‐resistant (*n* = 5) prostate cancer tumor specimens for proteomic, lipidomics, palmitoyl–proteomics analysis. B) volcano plot and density plot of proteomic analysis. C) KEGG and GO pathway enrichment analysis of differentially expressed proteins identified in proteomics. D) lipid metabolomic analysis of enzalutamide‐sensitive (*n* = 5) and ‐resistant (*n* = 5) prostate cancer tumor specimens. E) schematic diagram showing palmitic acid conversion to sphingomyelin (SM) and ceramide via palmitoyl‐CoA. F) volcano plot and density plot analysis of differentially expressed proteins in palmitoylome profiling. G) KEGG and GO pathway enrichment analysis of differentially palmitoylated proteins. H) C4‐2 and 22RV1 cells were treated with 2‐bromopalmitate (30 µm) for 24 h, followed by a serial dose of enzalutamide for 24 h. Cell viability was assessed using CCK‐8 assay. I) Transcriptome sequencing was performed on enzalutamide‐resistant and ‐sensitive C4‐2 cells to comparatively analyze the expression levels of ZDHHCs. J) Enzalutamide‐resistant C4‐2 and 22RV1 cells were infected with indicated shRNAs for 72 h. After puromycin selection, cells were treated with a serial dose of enzalutamide for 24 h. Cell viability was assessed using CCK‐8 assay. K) Enzalutamide‐resistant C4‐2 or 22RV1 cells were infected with the indicated shRNAs for 72 h. After puromycin selection, cells were treated with enzalutamide or vehicle for 24 h, followed by colony formation assays. Data represent mean ± SD (*n* = 3). **, *p* < 0.01; ***, *p* < 0.001. L) ZDHHC2‐knockout (ZDHHC2‐KO) mice were generated and treated with or without enzalutamide (10 mg/Kg). Representative images and mass of prostate tumors are shown. Data represent mean ± SD (*n* = 3). *, *p* < 0.05; **, *p* < 0.01. M) molecular structure of TTZ1, a ZDHHC2 inhibitor.

To investigate the mechanisms underlying enzalutamide resistance, we conducted multi‐omics analyses, including proteomics and metabolomics, in prostate cancer obtained from five enzalutamide‐sensitive and five enzalutamide‐resistant patients (**Figure** **1** **A)**. Our initial analysis of the proteomics data revealed highly overlapping density plots, indicating minimal differences in protein expression patterns between the two groups (Figure 1B). Kyoto Encyclopedia of Genes and Genomes (KEGG) and Gene Ontology (GO) enrichment analyses of the differentially expressed proteins revealed functional enrichment in lipid metabolism (Figure 1C; Table S5, Supporting Information). Considering the crucial role of lipid metabolism in CRPC progression and enzalutamide resistance,^[^ ^27^, ^28^, ^29^ ^]^ lipid metabolomics analysis was carried out. Our analysis revealed distinct sphingomyelin metabolism between enzalutamide‐resistant and ‐sensitive groups, while no differences were observed in other phospholipid (Figure 1D). To investigate whether sphingomyelin metabolism influences enzalutamide sensitivity, cell lines were treated with myriocin, the *de novo* synthesis of sphingolipid inhibitor.^[^ ^30^ ^]^ However, myriocin only slightly increased sensitivity to enzalutamide (Figure S1A, Supporting Information). It is noteworthy that palmitic acid serves as the initial metabolite for sphingomyelin synthesis (Figure 1E), and palmitic acid has been reported to play an important role in cancer progression.^[^ ^31^ ^]^ Thus, we hypothesize that palmitic acid may play a critical role in enzalutamide resistance. However, the lipid metabolomics analysis did not detect palmitic acid. So, the palmitic acid level between enzalutamide‐sensitive and resistant patient samples was compared, and the palmitic acid level was significantly higher in enzalutamide‐resistant patient prostate cancer samples (Figure S1B, Supporting Information). To simulate clinical enzalutamide resistance, we treated AR‐positive CRPC cell lines (C4‐2 and 22Rv1 cells) with enzalutamide or vehicle (DMSO) to establish enzalutamide‐resistant cell lines (Figure S1C, Supporting Information). And the palmitic acid level was increased in enzalutamide‐resistant cell lines, which matches the result of patient samples, indicating the important role of palmitic acid in enzalutamide resistance (Figure S1D, Supporting Information). Also, treatment of palmitic acid decreased the sensitivity of enzalutamide in cell lines (Figure S1E, Supporting Information). As the direct donor for protein S‐palmitoylation modification, altered palmitic acid levels may influence the protein palmitoylation status, thereby influencing cellular fate regulation.^[^ ^17^ ^]^ Consequently, we performed palmitoyl–proteomics analysis, where the irregular density plots demonstrated significant differences in palmitoylation modification patterns between enzalutamide‐resistant and ‐sensitive groups (Figure 1E,F). Proteins with significantly altered palmitoylation levels were enriched in pathways related to cancer, ferroptosis, cell growth, and fatty acid oxidation (Figure 1G; Table S6, Supporting Information). As expected, treatment with the palmitoylation inhibitor (2‐BP) increased sensitivity to enzalutamide both in vitro (Figure 1H) and in enzalutamide‐resistant patient‐derived tumor xenograft (PDX) models (Figure S1F, Supporting Information) significantly. Transcriptome sequencing data from a previous study were analyzed to compare the expression levels of ZDHHCs between enzalutamide‐resistant and enzalutamide‐sensitive CRPC cells.^[^ ^32^ ^]^ We found that ZDHHC2 exhibited the most significant change, substantially increasing in the enzalutamide‐resistant group (Figure 1I). Comparative analysis revealed a significant upregulation of ZDHHC2 in both enzalutamide‐resistant patient samples and cell lines (Figure S1G, Supporting Information). ZDHHC2 knockdown partially reversed enzalutamide resistance (Figure 1J,K; Figure S1H–J, Supporting Information), while ZDHHC2 overexpression promoted enzalutamide resistance in both enzalutamide‐resistant and sensitive cell lines (Figure S1K–N, Supporting Information). Furthermore, knockdown of ZDHHC2 could reverse the enzalutamide resistance induced by palmitic acid treatment, suggesting that palmitic acid promotes enzalutamide resistance through ZD2 (Figure S1O, Supporting Information). In enzalutamide‐sensitive C4‐2 cell‐derived xenograft (CDX) model mice, ZDHHC2 also promoted enzalutamide resistance (Figure S1P, Supporting Information). Notably, expression of an enzymatically dead ZDHHC2 mutant (C129A) failed to confer enzalutamide resistance,^[^ ^33^, ^34^, ^35^ ^]^ indicating that ZDHHC2‐mediated resistance is strictly dependent on its enzymatic activity (Figure S1K,M, Supporting Information). To elucidate the role of ZDHHC2 in the enzalutamide response in spontaneous prostate cancer models, we established a ZDHHC2 knockout (KO) mouse model through CRISPR/Cas‐mediated genome engineering (Figure S2A, Supporting Information) and verified ZDHHC2 KO via PCR and western blotting (Figure S2B–D, Supporting Information). The plasmid mixture was injected into the prostate to induce spontaneous prostate cancer at 6 weeks of age, and the mice were treated with enzalutamide or vehicle for 2 weeks. After 2 weeks of treatment, all mice were sacrificed, and their prostates were collected, weighed, and statistically analyzed (Figure S2E,F, Supporting Information). ZDHHC2 knockout significantly limited tumor growth and enhanced the enzalutamide sensitivity (Figure 1L). Additionally, the ZDHHC2‐specific palmitoylation inhibitor tetrazole‐1 (TTZ1) was synthesized as previously reported (Figure 1M; Figure S2G, Supporting Information).^[^ ^26^ ^]^ Treatment with TTZ1 significantly enhanced enzalutamide sensitivity in both enzalutamide‐sensitive (Figure S2H,I, Supporting Information) and enzalutamide‐resistant (Figure S2J, Supporting Information) CRPC cell lines. However, in the case of ZDHHC2 knockout, TTZ1 no longer affected the enzalutamide sensitivity (Figure S2K, Supporting Information). Furthermore, TTZ1 significantly enhanced the treatment effect of enzalutamide in enzalutamide‐resistant PDX models (Figure S2L, Supporting Information). Furthermore, to assess the toxicity of the combined treatment of TTZ1 and enzalutamide in vivo, we measured the indicators, including body weight, liver function, and kidney function of the mice. We found that the combination of enzalutamide and TTZ1 did not cause any changes in these indicators, suggesting a relatively high level of safety in vivo (Figure S2M,N, Supporting Information). Together, our results suggest that ZDHHC2 plays a key role in enzalutamide resistance through its palmitoylation function.

### 2.2 ZDHHC2 is Transcriptionally Upregulated by FOXA1/CXXC5/TET2 Complex in Enzalutamide‐Resistant CRPC cells

**Figure 2:**
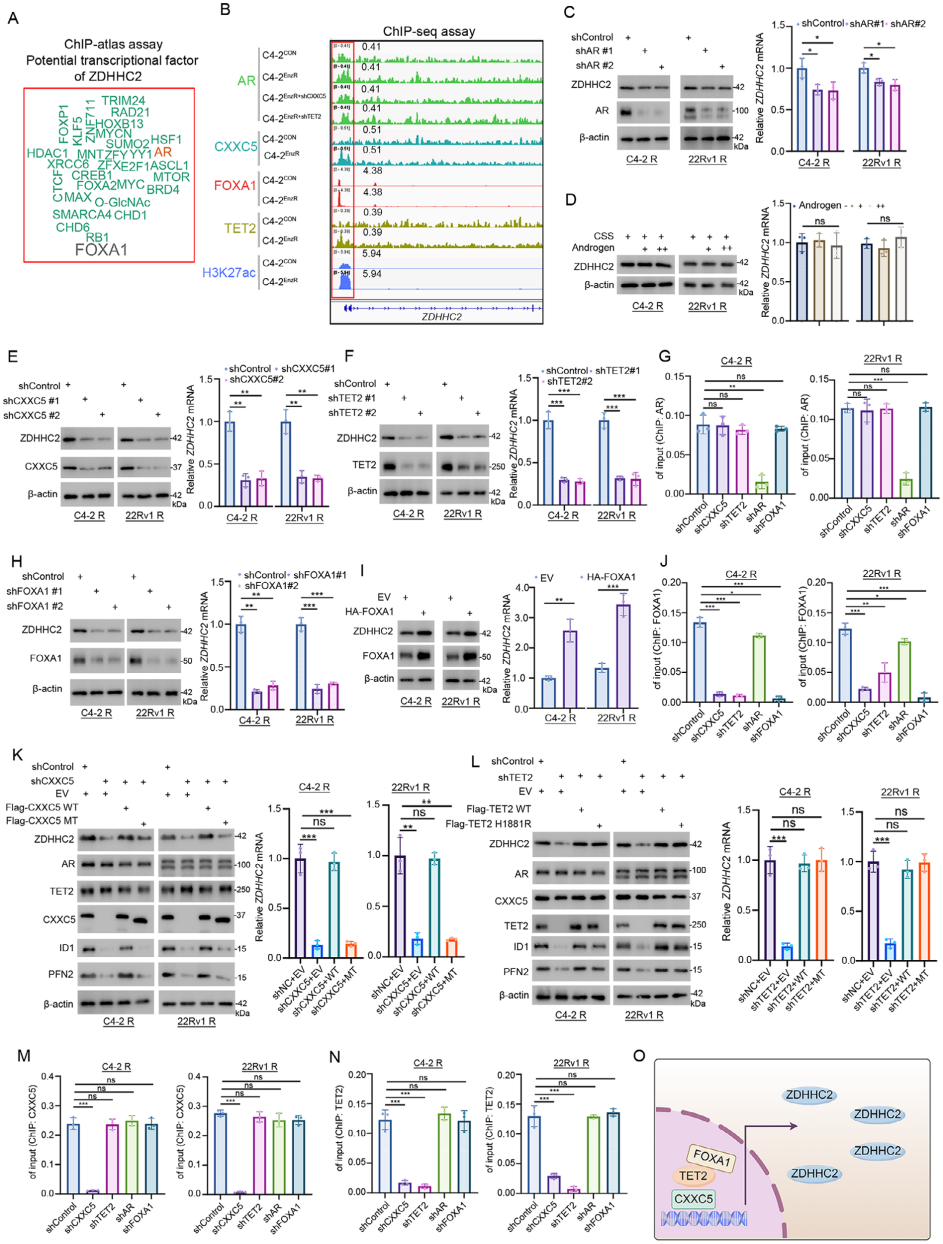
ZDHHC2 is transcriptionally upregulated by FOXA1/CXXC5/TET2 complex in enzalutamide‐resistant CRPC cells.A) Identification of potential transcription factors for ZDHHC2 via ChIP‐Atlas assay. B) ChIP‐seq analysis of ZDHHC2 promoter‐binding proteins in control and enzalutamide‐resistant C4‐2 cells. C) Enzalutamide‐resistant C4‐2 and 22Rv1 cells were transfected with AR shRNAs for 72 h. After puromycin selection, cells were harvested for Western blot and RT‐qPCR analysis of ZDHHC2 expression. Data represent mean ± SD (*n* = 3). *, *p* < 0.05. D) enzalutamide‐resistant C4‐2 and 22Rv1 cells were treated with charcoal‐stripped serum and androgen (0, 1, 2 µm) for 7 days. Data represent mean ± SD (*n* = 3). ns, not significant. E) Enzalutamide‐resistant C4‐2 and 22Rv1 cells were transfected with CXXC5 shRNAs for 72 h. After puromycin selection, cells were harvested for Western blot and RT‐qPCR analysis of ZDHHC2 expression. Data represent mean ± SD (*n* = 3). **, *p* < 0.01. F) Enzalutamide‐resistant C4‐2 and 22Rv1 cells were transfected with TET2 shRNAs for 72 h. After puromycin selection, cells were harvested for Western blot and RT‐qPCR analysis of ZDHHC2 expression. Data represent mean ± SD (*n* = 3). ***, *p* < 0.001. G) ChIP‐qPCR analysis of AR occupancy at genomic loci of ZDHHC2 in enzalutamide‐resistant C4‐2 and 22Rv1 cells transfected with control, CXXC5‐, TET2‐, AR‐, or FOXA1‐specific shRNAs. Data represent mean ± SD (*n* = 3). ns, not significant; **, *p* < 0.01; ***, *p* < 0.001. H) Enzalutamide‐resistant C4‐2 and 22Rv1 cells were transfected with control or FOXA1 shRNAs for 72 h. After puromycin selection, cells were harvested for Western blot and RT‐qPCR analysis of ZDHHC2 expression. Data represent mean ± SD (*n* = 3). **, *p* < 0.01; ***, *p* < 0.001. I) Enzalutamide‐resistant C4‐2 and 22Rv1 cells were transfected with an empty vector (EV) or FOXA1 overexpression plasmid. And cells were harvested for Western blot and RT‐qPCR analysis of ZDHHC2 expression. Data represent mean ± SD (*n* = 3). **, *p* < 0.01; ***, *p* < 0.001. J) ChIP‐qPCR analysis of FOXA1 occupancy at genomic loci of ZDHHC2 in enzalutamide‐resistant C4‐2 and 22Rv1 cells transfected with control, CXXC5‐, TET2‐, AR‐, or FOXA1‐specific shRNAs. Data represent mean ± SD (*n* = 3). ns, not significant; *, *p* < 0.05; **, *p* < 0.01; ***, *p* < 0.001. K,L) enzalutamide‐resistant C4‐2 and 22Rv1 cells were infected with indicated shRNAs or plasmids, followed by Western blot and RT‐qPCR analysis. ns, not significant; **, *p* < 0.01; ***, *p* < 0.001. M,N) ChIP‐qPCR analysis of CXXC5 (M) or TET2 (N) occupancy at genomic loci of ZDHHC2 in enzalutamide‐resistant C4‐2 and 22Rv1 cells transfected with control, CXXC5‐, TET2‐, AR‐, or FOXA1‐specific shRNAs. Data represent mean ± SD (*n* = 3). ns, not significant; ***, *p* < 0.001. O) schematic model: ZDHHC2 upregulation in enzalutamide‐resistant CRPC cells is mediated by a complex composed of CXXC5, TET2 And FOXA1 (By Figdraw).

Despite the significant upregulation of ZDHHC2 observed in the enzalutamide‐resistant group, the underlying mechanisms remain to be elucidated. Notably, the level of ZDHHC2 mRNA was increased in enzalutamide‐resistant CRPC cells (Figure 1I). Various potential transcription factors regulating ZDHHC2, including FOXA1 or AR, were screened by a ChIP‐Atlas (**Figure** **2**A). AR pathway is an important target of enzalutamide.^[^ ^36^ ^]^ FOXA1 enables AR to combine with chromatin by acting as a pioneer factor, and enzalutamide treatment can induce FOXA1 reprogramming from inactive chromatin sites to active cis‐regulatory elements.^[^ ^37^ ^]^ To investigate the regulation of ZDHHC2 expression, ChIP‐seq from our previous study was analyzed.^[^ ^32^ ^]^ We found that AR protein occupancy at the promoters and/or potential enhancers of ZDHHC2 showed no significant difference between enzalutamide‐resistant cells and control cells, but FOXA1 showed higher occupancy (Figure 2B). However, AR knockdown could partially decrease ZDHHC2 expression (Figure 2C), and the regulation of ZDHHC2 expression was independent of androgen (Figure 2D). There is a broad consensus that the expression of androgen response element‐dependent canonical AR target genes (such as KLK3) is suppressed in AR‐targeted therapy‐resistant CRPC.^[^ ^32^, ^38^, ^39^ ^]^ However, ZDHHC2 was overexpressed in enzalutamide‐resistant tissues and cells (Figure 1I; Figure S3A,B, Supporting Information), indicating that transcriptional regulation of ZDHHC2 differs from the canonical AR pattern. In our previous study, we identified a noncanonical AR function in which AR enhances the expression of a series of genes highly enriched with CpG islands (such as ID1 and PFN2) by forming a complex with CXXC5 and TET2; in addition, histone H3 lysine 27 (H3K27ac) levels in these genes were significantly increased in enzalutamide‐resistant cells.^[^ ^32^ ^]^ To explore whether ZDHHC2 is regulated by a similar mechanism, knockdown of CXXC5 and TET2 was performed, and both decreased the expression of ZDHHC2 significantly (Figure 2E,F). Meanwhile, CXXC5 and TET2 performed higher occupancy at ZDHHC2 promoters in enzalutamide‐resistant cells (Figure 2B). However, results of ChIP‐seq and ChIP‐qPCR showed that knockdown of CXXC5 or TET2 could not influence AR occupancy at ZDHHC2 promoter, indicating a different regulation mechanism form AR/CXXC5/TET2 complex (Figure 2B,G). Next, whether FOXA1 regulates ZDHHC2 expression was explored. Both knockdown and knockout of FOXA1 significantly decreased ZDHHC2 expression, while overexpression of FOXA1 increased ZDHHC2 expression (Figure 2H,I; Figure S3C,D, Supporting Information). Meanwhile, knockdown of CXXC5 or TET2 markedly reduced FOXA1 occupancy at ZDHHC2 promoter (Figure 2J), indicating the formation of FOXA1/CXXC5/TET2 complex. After CXXC5 knockdown, the import of wild‐type CXXC5 restored ZDHHC2 expression. However, the import of TET2 binding‐deficient mutant CXXC5 (1‐250) showed no effect^[^ ^32^ ^]^ (Figure 2K). Both wild‐type and catalytic activity‐deficient mutant (H1881R) TET2^[^ ^32^ ^]^ reversed the decrease in ZDHHC2 expression resulting from TET2 knockdown, proving that TET2 mediates ZDHHC2 expression independent of catalytic activity (Figure 2L). Additionally, knockdown of CXXC5 decreased TET2 occupancy at ZDHHC2 promoter (Figure 2M,N). And knockdown of CXXC5, TET2, or FOXA1 decreased the level of H3K27ac at ZDHHC2 stronger than AR knockdown, indicating that FOXA1/CXXC5/TET2 complex plays a more important role in ZDHHC2 expression regulation than AR (Figure S3E,F, Supporting Information). Taken together, these findings suggest that the overexpression of ZDHHC2 in enzalutamide‐resistant CRPC cells is mediated by the complex composed of FOXA1, TET2, and CXXC5 (Figure 2O).

### 2.3 ZDHHC2 Promotes Enzalutamide Resistance by Inhibiting Lipid Peroxide Production and Ferroptosis

**Figure 3:**
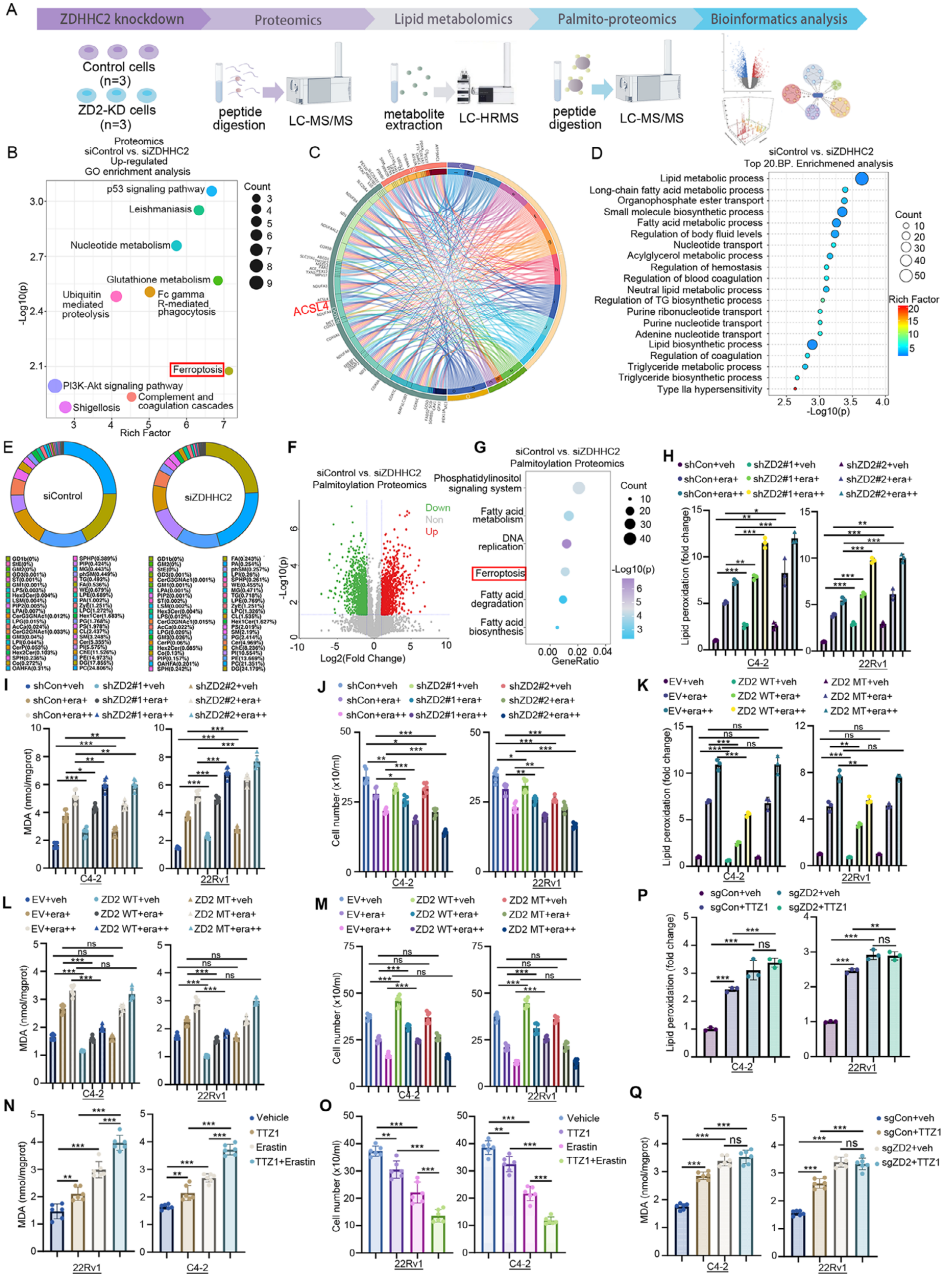
ZDHHC2 promotes enzalutamide resistance by inhibiting lipid peroxide production and ferroptosis. A) flowchart illustrating the prostate cancer cell lines for proteomic, lipidomics, palmitoyl–proteomics analysis. B,C) C4‐2 cells with or without ZDHHC2 knockdown were harvested for proteomic analysis. Upregulated differentially expressed proteins underwent Gene Ontology (GO) enrichment analysis. D) C4‐2 cells with or without ZDHHC2 knockdown were collected for lipid metabolomics, followed by enrichment analysis of the top 20 differentially abundant lipids. E) lipidomic profiling was performed in C4‐2 cells with or without ZDHHC2 knockdown to assess changes in lipid composition. F,G) Palmitoylation proteomics was conducted in C4‐2 cells with or without ZDHHC2 knockdown. Differentially palmitoylated proteins were visualized in a volcano plot (F) and analyzed via pathway enrichment (G). H–J) 22Rv1 and C4‐2 cells were infected with the indicated shRNAs for 72 h. After puromycin selection, cells were treated with or without Erastin (10 or 20 µm) for 24 h, followed by analysis of lipid peroxidation (H), malondialdehyde (MDA) levels (I), and cell counting (J). Data represent mean ± SD (*n* = 3 or 6). *, *p* < 0.05; **, *p* < 0.01; ***, *p* < 0.001. K–M) 22Rv1 and C4‐2 cells were transfected with the indicated plasmids for 24 h, then treated with or without Erastin (10 or 20 µm) for 24 h and analyzed for lipid peroxidation (K), MDA (L), and cell counting (M). Data represent mean ± SD (*n* = 3 or 6). ns, not significant; *, *p* < 0.05; **, *p* < 0.01; ***, *p* < 0.001. N‐O) 22Rv1 and C4‐2 cells were treated by TTZ1 (10 µm) with or without Erastin for 24 h and analyzed for MDA (N) assay and cell counting assay (O). **, *p* < 0.01; ***, *p* < 0.001. P,Q) Endogenous ZDHHC2 was knocked out in C4‐2 and 22Rv1 cells using CRISPR/Cas9, and cells were treated with or without TTZ1 (10 µm) for 24 h and analyzed for lipid peroxidation (P) and MDA (Q). Data represent mean ± SD (*n* = 3 or 6). ns, not significant; **, *p* < 0.01; ***, *p* < 0.001.

Although ZDHHC2 promotes enzalutamide resistance in prostate cancer, the underlying mechanisms remain to be elucidated, so multi‐omics analyses were performed with control and ZDHHC2 knockdown C4‐2 cell lines (**Figure** **3** **A)**. Proteomic analysis between control and ZDHHC2 knockdown cells demonstrated that ZDHHC2 is involved in both ferroptosis and lipid metabolism, while significantly downregulating the expression of ACSL4, a critical gene in ferroptosis regulation (Figure 3B–D; Figure S4A, Table S7, Supporting Information). Interestingly, this result matches the proteomics and palmitoyl‐proteomics analysis between enzalutamide‐resistant and sensitive patient samples, proving the crucial role of ferroptosis in enzalutamide resistance (Figure 1C,G). Our lipidomics analysis revealed that ZDHHC2 knockdown significantly altered the lipid metabolite composition in prostate cancer cells, providing compelling evidence for the pivotal role of ZDHHC2 in mediating enzalutamide resistance through lipid metabolic reprogramming (Figure 3E; Figure S4B,C, Supporting Information). Given the potential role of ZDHHC2 in mediating the transfer of dysregulated palmitate‐derived palmitoyl‐CoA to target proteins for post‐translational modification, we performed palmitoyl‐proteomics analysis, which demonstrated that ZDHHC2 significantly alters the palmitoylation status of multiple proteins involved in both lipid metabolism and ferroptosis pathways (Figure 3F,G; Table S8, Supporting Information). Ferroptosis is a form of regulated necrotic cell death caused by the overwhelming accumulation of membrane lipid peroxides and is closely related to the phosphatidylinositol signaling system.^[^ ^40^, ^41^, ^42^ ^]^ A series of studies have identified ferroptosis as an emerging therapeutic target for prostate cancer and enzalutamide resistance.^[^ ^28^, ^29^, ^43^ ^]^ To further clarify the role of ferroptosis in enzalutamide resistance, both sensitive and resistant cell lines were treated with enzalutamide for ferroptosis indicator detecting. In sensitive cell lines, enzalutamide treatment significantly elevated the level of both lipid peroxidation (Figure S4D, Supporting Information) and malondialdehyde (MDA) (Figure S4E, Supporting Information), and reduced cell number (Figure S4F, Supporting Information). However, these indicators showed no significant change in resistant cell lines after enzalutamide treatment, indicating the key role of ferroptosis inhibition in enzalutamide resistance (Figure S4D–F, Supporting Information). We propose that ZDHHC2‐mediated suppression of ferroptosis contributes to the development of enzalutamide resistance in prostate cancer. With or without erastin treatment, ZDHHC2 knockdown significantly increased the level of lipid peroxidation (Figure 3H), lipid reactive oxygen species (ROS) (Figure S4G, Supporting Information), and the level of malondialdehyde (MDA) (Figure 3I), while reduced cell number (Figure 3J), proving that ZDHHC2 knockdown promoted ferroptosis induced by erastin of different concentration. In contrast, the overexpression of wild‐type ZDHHC2 instead of the enzymatically dead mutant reduced lipid peroxidation (Figure 3K), lipid ROS (Figure S4H, Supporting Information) and MDA (Figure 3L) levels, while increased cell numbers (Figure 3M) and maintained the morphology of mitochondria under treatment with the ferroptosis inducer erastin (Figure S4I, Supporting Information), indicating that ZDHHC2 inhibits ferroptosis by its palmitoylation function to maintain CRPC cell survival. The effects of TTZ1 treatment were like those of ZDHHC2 knockdown on lipid peroxidation, MDA content, and cell number and promoted mitochondrial damage (Figure 3N,O; Figure S4J, Supporting Information). Furthermore, after ZDHHC2 knockout, TTZ1 no longer affected lipid peroxidation (Figure 3P), MDA (Figure 3Q) content, or cell number (Figure S4K, Supporting Information), demonstrating that TTZ1 specifically targets ZDHHC2. CDX models were constructed with the enzalutamide‐sensitive or ‐resistant C4‐2 cell lines, and were treated with enzalutamide or liproxstatin‐1 (lip‐1, which is a widely recognized stable ferroptosis inhibitor in vivo).^[^ ^44^, ^45^ ^]^ In the sensitive CDX group, lip‐1 could attenuate the tumor inhibition of enzalutamide, suggesting the important role of ferroptosis in enzalutamide therapeutic effect. However, in the resistant group, this effect of enzalutamide and lip‐1 was not significantly observed (Figure S5A, Supporting Information). Together, these results suggest that ZDHHC2 promotes enzalutamide resistance by inhibiting ferroptosis via its palmitoylation function. Additionally, to demonstrate that CXXC5/FOXA1/TET2 complex of the noncanonical pathway promotes enzalutamide resistance by inhibiting ferroptosis, these genes were overexpressed in enzalutamide‐sensitive cell lines (Figure S5B, Supporting Information) and knockdown in enzalutamide‐resistant cell lines. As we expected, overexpression of FOXA1, CXXC5, or TET2 inhibited ferroptosis (Figure S5C–E, Supporting Information), while knockdown of FOXA1, CXXC5, or TET2 promoted ferroptosis (Figure S5F–H, Supporting Information).

### 2.4 ZDHHC2 Promotes the Ubiquitin‒Proteasome Degradation of ACSL4 through USP19

**Figure 4:**
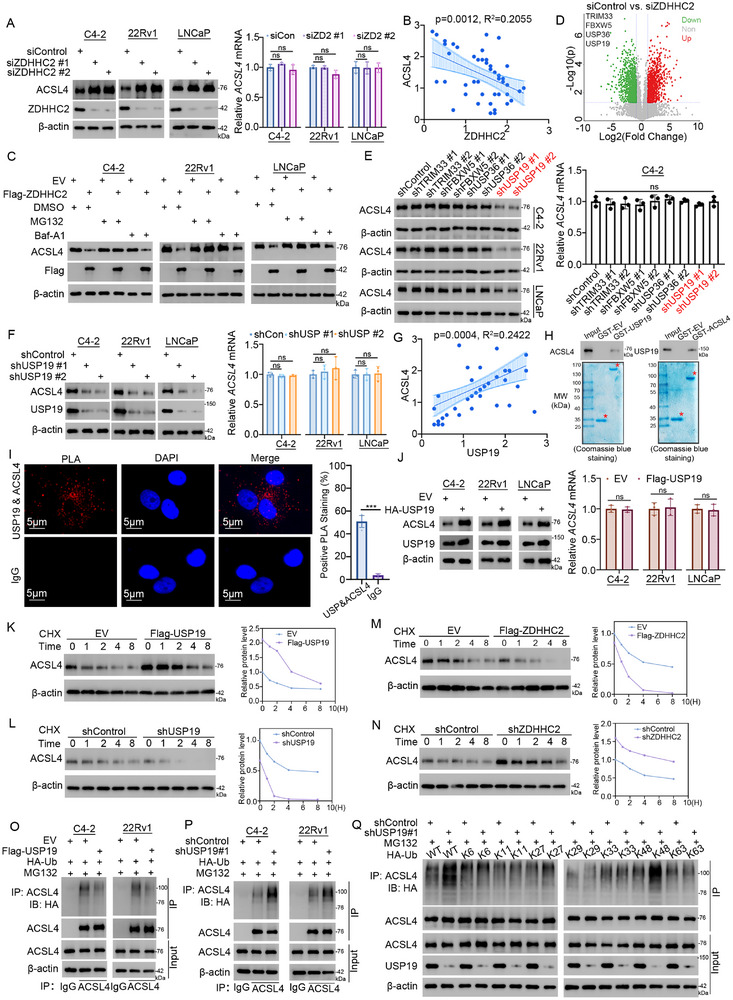
ZDHHC2 promotes the ubiquitin‒proteasome degradation of ACSL4 through USP19. A) C4‐2, 22Rv1, and LNCaP cells were transfected with the indicated siRNAs for 48 h and harvested for Western blot and RT‐qPCR analysis. Data represent mean ± SD (*n* = 3). ns, not significant. B) Immunohistochemical (IHC) staining was performed on prostate cancer tissue microarrays using ZDHHC2 and ACSL4 antibodies. The correlation between ZDHHC2 and ACSL4 expression is shown in panel B (*p* = 0.0012). C) C4‐2, 22Rv1, and LNCaP cells were transfected with the indicated plasmids for 24 h and treated with MG132 or bafilomycin A1 (Baf‐A1), followed by Western blot analysis. D) Volcano plot displaying proteins with different palmitoylation levels from palmitoylation proteomics analysis of cells with or without ZDHHC2 knockdown. E) C4‐2 cells were infected with indicated shRNAs for 72 h. After puromycin selection, cells were harvested for Western blot and RT‐qPCR analysis. Data represent mean ± SD (*n* = 3). ns, not significant. F) C4‐2, 22Rv1, and LNCaP cells were infected with indicated shRNAs for 72 h. After puromycin selection, cells were analyzed by Western blot and RT‐qPCR analysis. Data represent mean ± SD (*n* = 3). ns, not significant. G) IHC staining of prostate cancer tissue microarray using USP19 and ACSL4 antibodies. Correlation between USP19 and ACSL4 expression is shown (*p* = 0.0004). H) GST pull‐down assay performed with recombinant USP19 or ACSL4 proteins. I) Proximity ligation assay (PLA) in 22Rv1 cells using the indicated antibodies. J) C4‐2, 22Rv1, and LNCaP cells were transfected with the indicated plasmids for 24 h and analyzed by Western blot and RT‐qPCR analysis. Data represent mean ± SD (*n* = 3). ns, not significant. K–N) 22Rv1 cells transfected with indicated plasmids (K and M) or shRNAs (L and N) were treated with cycloheximide (CHX) and collected at specified time points for Western blot analysis of protein stability. O,P) C4‐2 and 22Rv1 cells transfected with indicated plasmids (O) or shRNAs (P) were treated with MG132 and harvested for co‐IP and Western blot analysis. Q) 22Rv1 cells transfected with indicated shRNAs were treated with MG132 and collected for co‐IP and Western blot analysis.

We subsequently investigated the underlying mechanisms by which ZDHHC2 suppresses ferroptosis. Proteomic analysis revealed that after ZDHHC2 knockdown, the key driver of lipid peroxide production and ferroptosis, ACSL4, increased significantly (Figure 3C). Recent studies have identified that ZDHHC family members, particularly ZDHHC8 and ZDHHC20, play regulatory roles in ferroptosis through mediating protein palmitoylation, with GPX4 serving as a key substrate.^[^ ^23^, ^24^ ^]^ However, the relationship between ZDHHCs and ferroptosis is mainly focused on lipid peroxide clearance proteins.^[^ ^46^ ^]^ There is limited research on the relationship between ZDHHCs and proteins related to lipid peroxide production. ACSL4 facilitates the synthesis of polyunsaturated phospholipids (PUFA‐PLs) that are particularly vulnerable to radical attack, thereby promoting lipid peroxidation and ultimately inducing ferroptosis.^[^ ^47^ ^]^ We showed that ZDHHC2 knockdown increased ACSL4 at the protein level but not at the mRNA level (**Figure** **4**A), and ZDHHC2 overexpression decreased ACSL4 only at the protein level (Figure S6A, Supporting Information), indicating that ZDHHC2 regulates ACSL4 by altering protein stability. Immunohistochemical (IHC) staining of a prostate cancer tissue microarray revealed a negative correlation between the protein levels of ACSL4 and ZDHHC2 (Figure 4B; Figure S6B, Supporting Information). To delineate how ZDHHC2 modulates ACSL4, cell lines overexpressing ZDHHC2 were treated with the ubiquitin–proteasome system inhibitor MG132 or the autophagosome–lysosome system inhibitor Baf‐A1. Only MG132 reversed ACSL4 degradation caused by ZDHHC2 overexpression (Figure 4C), indicating that ZDHHC2 regulates ACSL4 in a ubiquitin‐dependent manner. Although ZDHHC2 has not been reported to have functions in the ubiquitin–proteasome system, we identified four ubiquitin–proteasome associated proteins‐TRIM33, FBXW5, USP36, and USP19‐among those whose palmitoylation levels were significantly altered following ZDHHC2 knockdown (Figure 4D). Knockdown experiments revealed that only USP19 affected ACSL4 at the protein level but not at the mRNA level (Figure 4E,F). Furthermore, the prostate cancer tissue microarray revealed a positive correlation between the protein levels of ACSL4 and USP19 (Figure 4G; Figure S6C, Supporting Information). Coimmunoprecipitation was performed using three CRPC cell lines to demonstrate the endogenous interaction between USP19 and ACSL4 (Figure S6D, Supporting Information). GST pull‐down confirmed a direct physical combination with the recombinant proteins (Figure 4H). A proximity ligation assay (PLA) further verified their spatial proximity interaction (Figure 4I). In addition, the overexpression of USP19 increased ACSL4 protein levels without affecting ACSL4 mRNA levels (Figure 4J). Next, we found that the overexpression of USP19 extended the half‐life of the ACSL4 protein, whereas USP19 knockdown shortened it (Figure 4K,L). In contrast, ZDHHC2 had the opposite effect on the half‐life of the ACSL4 protein (Figure 4M,N). Moreover, the overexpression of USP19 decreased the polyubiquitination of ACSL4, whereas USP19 knockdown increased it (Figure 4O,P). Notably, USP19 specifically inhibited the K48‐linked ubiquitination of ACSL4 (Figure 4Q), confirming that USP19 is a stabilizing deubiquitinase of ACSL4.

### 2.5 ZDHHC2 Mediates the Palmitoylation of USP19

**Figure 5:**
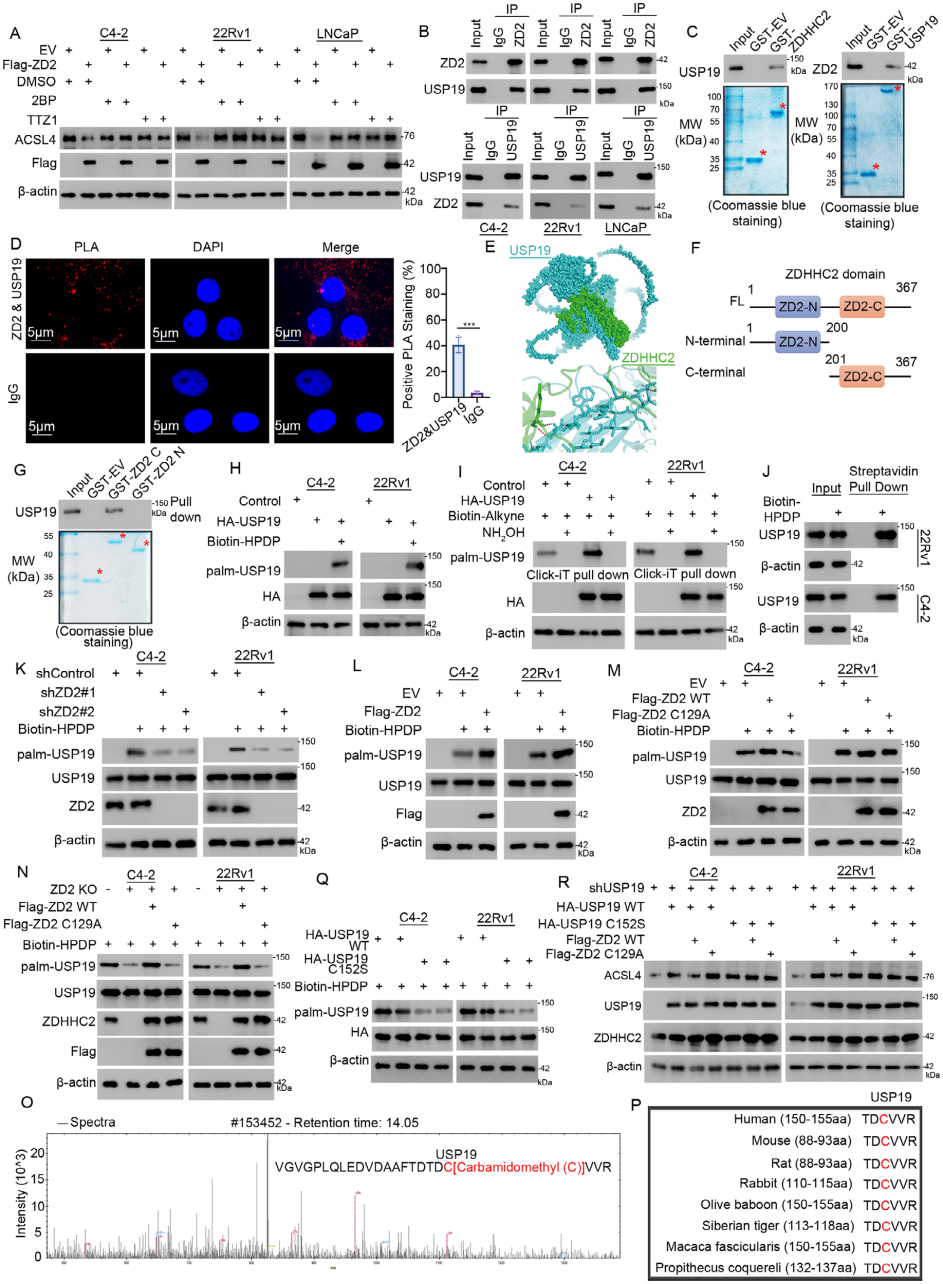
ZDHHC2 mediates the palmitoylation of USP19. A) C4‐2, 22Rv1, and LNCaP cells were transfected with the indicated plasmids for 24 h, followed by treatment with 2‐bromopalmitate (2BP, 30 µm) or TTZ1 (10 µm) for 24 h before Western blot analysis. B) C4‐2, 22Rv1, and LNCaP cell lysates were subjected to co‐immunoprecipitation (co‐IP) using the specified antibodies. C) GST pull‐down assays were performed using recombinant USP19 or ZDHHC2 proteins. D) PLA was conducted in 22Rv1 cells with the indicated antibodies. Data represent mean ± SD from six replicates. ***, *p* <0.001. E) Molecular docking simulations were performed to model USP19‐ZDHHC2 interactions. F) schematic representation of ZDHHC2 domain architecture. G) GST pull‐down assays were conducted using full‐length ZDHHC2 or its N‐terminal/C‐terminal fragments. H) C4‐2 and 22Rv1 cells transfected with empty vector or HA‐USP19 plasmids for 24 h were analyzed by acyl‐biotinyl exchange (ABE) assay to detect USP19 palmitoylation. I) C4‐2 and 22Rv1 cells transfected with control or HA‐USP19 plasmids for 24 h were analyzed by Click‐iT palmitoylation assay. J) Endogenous USP19 palmitoylation levels in C4‐2 and 22Rv1 cells were assessed by ABE assay. K) C4‐2 and 22Rv1 cells were infected with the indicated shRNAs for 72 h. After puromycin selection, USP19 was labeled with Biotin‐HPDP for streptavidin pull‐down to analyze its palmitoylation levels. L,M) C4‐2 and 22Rv1 cells were transfected with empty vector or Flag‐ZDHHC2 plasmids for 24 h. ABE assay was performed to detect USP19 palmitoylation levels. N) endogenous ZDHHC2 was knocked out in C4‐2 and 22Rv1 cells using CRISPR/Cas9. ZDHHC2‐KO cells were then transfected with the indicated plasmids for 24 h. USP19 palmitoylation levels were assessed by ABE assay. O) peptide mapping of USP19 palmitoylation at the C152 site mediated by ZDHHC2. P) The amino acid sequence of USP19 palmitoylated by ZDHHC2 is evolutionarily conserved across species. Q) C4‐2 and 22Rv1 cells were transfected with the indicated plasmids for 24 h. USP19 palmitoylation was analyzed by ABE assay. R) C4‐2 and 22Rv1 cells were infected with shUSP19 for 72 h. After puromycin selection, cells were transfected with the indicated plasmids for 24 h and harvested for Western blot analysis.

Next, we explored whether ZDHHC2 regulates ACSL4 levels by palmitoylating USP19. Both the pan‐palmitoylation inhibitor 2‐BP and the ZDHHC2‐specific palmitoylation inhibitor TTZ1 reversed ACSL4 degradation after ZDHHC2 overexpression (**Figure** **5**A). Furthermore, the elevation effect of TTZ1 on the ACSL4 protein level was dose‐related. After ZDHHC2 knockout, TTZ1 showed no effect on ACSL4 protein level, suggesting that TTZ1 influences ACSL4 through ZDHHC2 (Figure S7A, Supporting Information). Coimmunoprecipitation confirmed the endogenous interaction between ZDHHC2 and USP19 (Figure 5B), GST pull‐down verified the direct physical interaction (Figure 5C), and PLA further demonstrated spatial proximity interactions (Figure 5D). Next, the binding region of USP19 to ZDHHC2 was analyzed via molecular docking (Figure 5E,F) and GST pull‐down assays (Figure 5G), and we found that USP19 binds to the C‐terminal region of ZDHHC2. Both exogenous and endogenous USP19 were demonstrated to undergo palmitoylation through employing the acyl‐biotinyl exchange (ABE) assay and click‐iT pulldown experimental approaches (Figure 5H–J). To confirm whether ZDHHC2 is the only ZDHHC enzyme that mediates the palmitoylation of USP19, a set of ZDHHC‐knockout cell lines was established using a CRISPR screen. Only ZDHHC2 knockout significantly abolished the palmitoylation of USP19 (Figure 5K; Figure S7B, Supporting Information). The overexpression of wild‐type ZDHHC2 but not the enzymatically dead mutant increased the palmitoylation level of USP19 (Figure 5L,M). The results of the knockdown and rescue experiments were similar (Figure 5N). Palmitoylation proteomics analysis revealed that cysteine (C) 152 is the palmitoylation site of USP19 (Figure 5O), and this site is highly conserved among different species (Figure 5P). After the mutation of 152C to serine (S), the palmitoylation level of USP19 significantly decreased (Figure 5Q). Additionally, the increase in ACSL4 protein caused by USP19 C152S mutant overexpression could not be reversed by ZDHHC2 overexpression (Figure 5R), which further confirmed that ZDHHC2 promotes ACSL4 degradation by palmitoylating USP19. Meanwhile, we constructed the mutant USP19 C152S with enzalutamide‐resistant cell lines. This mutation prevented USP19 from being palmitoylated by ZDHHC2 (Figure S7C, Supporting Information), and the ACSL4 protein level in the mutant was higher compared to the wild‐type (Figure S7D, Supporting Information). Besides, as we have demonstrated that palmitic acid treatment could reduce the enzalutamide sensitivity through ZDHHC2 (Figure S1E,O, Supporting Information), to clarify the molecular mechanism, we detected USP19 and ACSL4 after palmitic acid treatment. We found that palmitic acid elevated palmitoylation of USP19 (Figure S7E, Supporting Information) and reduced ACSL4 protein level (Figure S7F, Supporting Information), and these effects disappeared after ZDHHC2 knockout (Figure S7E,G, Supporting Information). To further verify the correlation between ZD2‐USP19‐ACSL4 axis and enzalutamide resistance, we detected USP19 and ACSL4 in enzalutamide‐sensitive and resistant cell lines. In the resistant cell lines, mRNA level of USP19 and ACSL4 remained unchanged (Figure S7H, Supporting Information), palmitoylation of USP19 was elevated, and ACSL4 protein was decreased (Figure S7I, Supporting Information).

### 2.6 The Palmitoylation of USP19 Weakens its Interaction with ACSL4 and Promotes Enzalutamide Resistance by Inhibiting Ferroptosis

**Figure 6:**
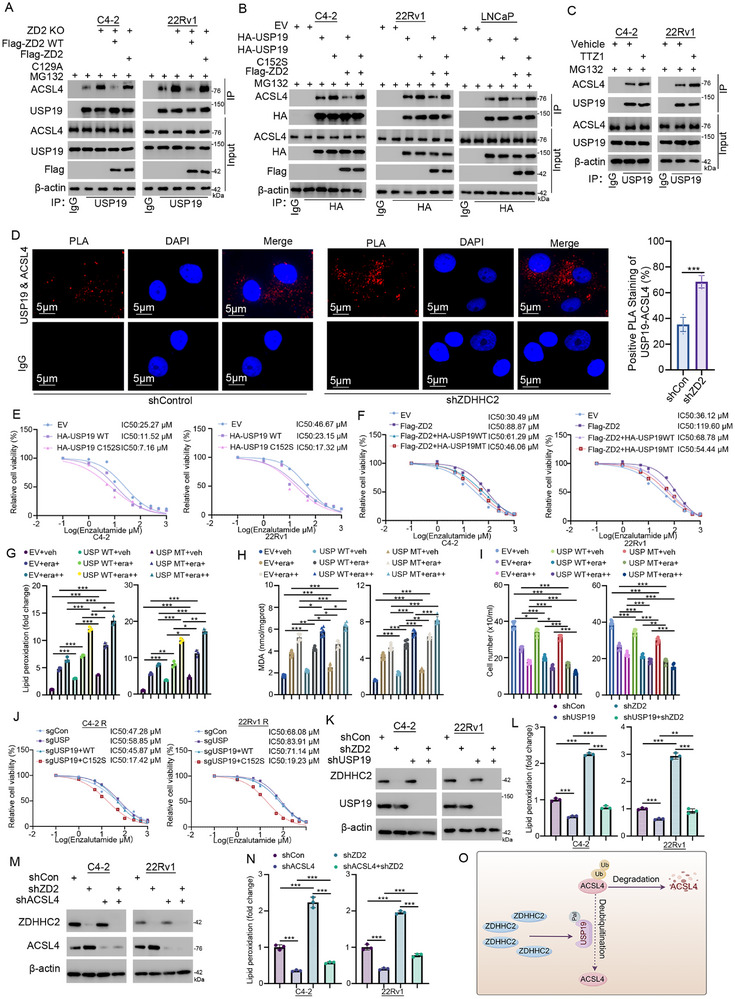
The palmitoylation of USP19 weakens its interaction with ACSL4 and promotes enzalutamide resistance by inhibiting ferroptosis.A) Endogenous ZDHHC2 was knocked out in C4‐2 and 22Rv1 cells with CRISPR/Cas9. The ZDHHC2‐knockout (ZDHHC2‐KO) cells were then transfected with the indicated plasmids for 24 h, treated with MG132, and harvested for co‐IP and Western blot analysis. B) C4‐2, 22Rv1, and LNCaP cells were transfected with the indicated plasmids for 24 h, treated with MG132, and were collected for co‐IP and Western blot analysis. C) C4‐2 and 22Rv1 cells were treated with or without TTZ1 and MG132 for 24 h before collection for co‐IP and Western blot analysis. D) PLA was performed in 22Rv1 cells using the indicated antibodies. Data represent mean ± SD from three replicates. ***, *p* < 0.001. E) Enzalutamide‐sensitive C4‐2 and 22Rv1 cells were transfected with the indicated plasmids for 24 h, then treated with increasing doses of enzalutamide for 24 h before CCK‐8 viability assays. F) C4‐2 and 22Rv1 cells transfected with the indicated plasmids for 24 h were treated with enzalutamide dose escalation for 24 h and assayed using CCK‐8. G–I) 22Rv1 and C4‐2 cells transfected with the indicated plasmids for 24 h were treated with or without Erastin (10 or 20 µm) for 24 h and analyzed for lipid peroxidation (G), MDA(H), and cell counting (I). *, *p* < 0.05; **, *p* < 0.01; ***, *p* < 0.001. J) Endogenous USP19 was knocked out in C4‐2 and 22Rv1 cells using CRISPR/Cas9. USP19‐KO cells were then transfected with the indicated plasmids for 24 h, then treated with increasing doses of enzalutamide for 24 h before CCK‐8 viability assays. K,L) 22Rv1 and C4‐2 cells were infected with the indicated shRNAs for 72 h (puromycin‐selected), western blot was performed for verification, and the lipid peroxidation level was measured. **, *p* < 0.01; ***, *p* < 0.001. M,N) 22Rv1 and C4‐2 cells were infected with indicated shRNAs for 72 h (puromycin‐selected), western blot was performed for verification, and the lipid peroxidation level was measured. ***, *p* < 0.001. O) schematic model: ZDHHC2‐mediated palmitoylation of USP19 weakens the interaction between USP19 and ACSL4 (By Figdraw).

**Figure 7:**
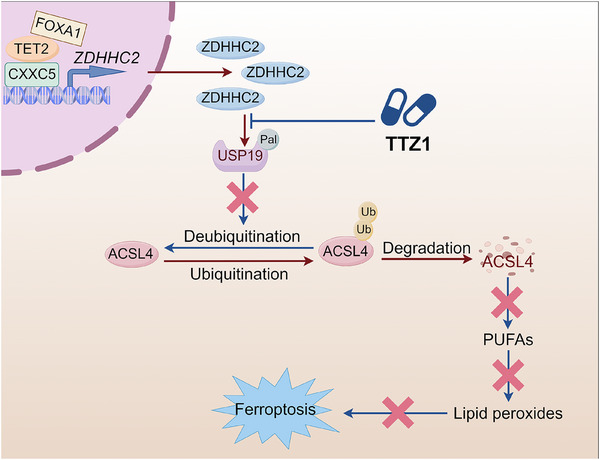
A schematic model demonstrates that ZDHHC2 is aberrantly overexpressed in CRPC via the FOXA1/CXXC5/TET2 complex and inhibits ferroptosis through the USP19/ACSL4 signaling axis. (By Figdraw).

To explore the effect of USP19 palmitoylation, we assessed the protein level of USP19 following ZDHHC2 knockdown; however, no change was observed. (Figure S8A, Supporting Information). Therefore, we propose that USP19 palmitoylation attenuates its interaction with ACSL4, thereby facilitating ACSL4 ubiquitination and subsequent degradation. This hypothesis was supported by co‐immunoprecipitation assays, which demonstrated that wild‐type ZDHHC2 overexpression significantly reduced the ACSL4‐USP19 protein–protein interaction (Figure 6A; Figure S8B, Supporting Information**),** but did not influence the binding between ACSL4 and the USP19 C152S mutant, which cannot be palmitoylated by ZDHHC2 (Figure 6B). In addition, treatment with TTZ1 also enhanced their combination (Figure 6C; Figure S8C, Supporting Information). Meanwhile, compared to enzalutamide‐sensitive cell lines, the ACSL4‐USP19 protein–protein interaction was weakened in resistant cell lines, which matches our hypothesis (Figure S8D, Supporting Information). PLA was performed to analyze the interaction between USP19 and ACSL4 after ZDHHC2 knockdown. ZDHHC2 knockdown significantly enhanced the interaction between USP19 and ACSL4 (Figure 6D). Taken together, these findings indicate that the palmitoylation mediated by ZDHHC2 at the C152 site of USP19 weakens its interaction with ACSL4, increases the degree of ubiquitination, and promotes the ubiquitin‒proteasome‐mediated degradation of ACSL4. Next, we explored the role of the ZDHHC2‐USP19‐ACSL4 axis in ferroptosis and enzalutamide resistance in CRPC. We found that the overexpression of both wild‐type and C152S mutant USP19 increased enzalutamide sensitivity (Figure 6E; Figure S8E,F, Supporting Information), whereas knockdown decreased enzalutamide sensitivity (Figure S8G,H, Supporting Information). Moreover, the overexpression of USP19 reversed enzalutamide resistance caused by ZDHHC2 overexpression (Figure 6F). Given that ZDHHC2 regulates ACSL4 through USP19, we subsequently investigated the functional impact of the ZDHHC2‐USP19 axis on ferroptosis. USP19 overexpression increased the levels of lipid peroxidation (Figure 6G), lipid ROS (Figure S8I, Supporting Information), and MDA (Figure 6H), while decreasing the cell numbers (Figure 6I). Furthermore, overexpression of the palmitoylation‐deficient USP19 C152S mutant significantly enhanced ferroptosis induction (Figure 6G–I). Meanwhile, the USP19 C152S mutant cell lines showed stronger indicators of ferroptosis, including higher lipid peroxidation (Figure S8J, Supporting Information), higher MDA (Figure S8K, Supporting Information), and lower cell numbers (Figure S8L, Supporting Information), and behaved more sensitive to enzalutamide than USP19 wild type (Figure 6J). Additionally, USP19 knockdown reversed the ferroptosis‐promoting effect of ZDHHC2 knockdown (Figure 6K; Figure S9A, Supporting Information), including decreases in the levels of lipid peroxidation (Figure 6L), lipid ROS (Figure S9B, Supporting Information) and MDA (Figure S9C, Supporting Information) and increases in the number of cells (Figure S9D, Supporting Information). And ACSL4 knockdown had a similar effect (Figure 6M,N; Figure S9E–H, Supporting Information). Finally, we explored the impact of ZDHHC2/USP19 overexpression on mitochondrial morphology. USP19 overexpression promoted mitochondrial damage, and the USP19 C152S mutant caused greater damage than did wild type USP19. Moreover, the overexpression of ZDHHC2 reversed only the mitochondrial damage caused by wild‐type USP19, not the damage caused by the USP19 C152S mutant (Figure S9I, Supporting Information). In brief, our results demonstrated that ZDHHC2 palmitoylates the deubiquitinase USP19, weakening the binding between USP19 and ACSL4, thereby promoting the ubiquitin‒proteasome degradation of ACSL4 (Figure 6O). Taken together, we identified ZDHHC2 as a novel target for enzalutamide resistance in CRPC. Mechanistically, ZDHHC2 overexpression is driven by the FOXA1/CXXC5/TET2 transcriptional complex, which subsequently suppresses ferroptosis through the USP19/ACSL4 regulatory axis (**Figure** **7**).

## 3 Discussion

Palmitic acid is a crucial precursor for the synthesis of sphingomyelin.^[^ ^48^ ^]^ We found that enzalutamide‐resistant prostate cancer is closely associated with phospholipid metabolism (specifically sphingomyelin rather than other phospholipid types), and that palmitic acid, a key substrate for sphingomyelin synthesis, is elevated in this context.^[^ ^48^ ^]^ High levels of palmitate activate prostate cancer cells and promote disease progression.^[^ ^49^ ^]^ As a substrate for palmitoylation, palmitic acid functions through palmitoyltransferases, which transfer palmitoyl groups to cysteine residues on target proteins.^[^ ^50^ ^]^ Some studies report that ZDHHC7 and ZDHHC21 enhance AR S‐palmitoylation, promoting prostate cancer progression and abiraterone resistance.^[^ ^51^ ^]^ Additionally, Wnt‐1 palmitoylation and subsequent pathway activation stabilize β‐catenin, representing a potential oncogenic mechanism in prostate cancer.^[^ ^52^ ^]^ Among all palmitoyl transferases, we found that only ZDHHC2 is significantly increased in prostate cancer during enzalutamide resistance. Our recent studies have shown that ZDHHC2 promotes sunitinib resistance in renal cell carcinoma by increasing the membrane localization of acylglycerol kinase (AGK).^[^ ^35^ ^]^ Similarly, we demonstrated that ZDHHC2 promotes enzalutamide resistance in prostate cancer cells, and mechanistically, we discovered that ZDHHC2 inhibits ferroptosis in prostate cancer cells.

Ferroptosis, an iron‐dependent form of regulated cell death triggered by the lethal accumulation of lipid peroxides on cell membranes, offers new hope for the treatment of malignant tumors.^[^ ^53^, ^54^, ^55^ ^]^ A recent study revealed that combining AR antagonists with ferroptosis inducers significantly suppresses the growth of AR+ prostate cancer.^[^ ^56^ ^]^ Moreover, enzalutamide reduces glutathione (GSH) production and induces ferroptosis, whereas AR‐Vs confer ferroptosis resistance in prostate cancer cells by upregulating SLC7A11 expression.^[^ ^28^ ^]^ Furthermore, PHGDH, a key enzyme in the serine synthesis pathway, inhibits ferroptosis and promotes enzalutamide resistance.^[^ ^29^ ^]^ These findings suggest that the AR‐mediated inhibition of ferroptosis leads to enzalutamide resistance, but the impact of FOXA1 has not been fully discussed. And here, we discovered a novel pathway improving enzalutamide resistance independent of AR. The formation of FOXA1/CXXC5/TET2 complex enhanced ZDHHC2 expression, indicating a new function of FOXA1, and provided a novel potential target to overcome enzalutamide resistance.

Recent studies showed that palmitoylation mediated by ZDHHC20 and ZDHHC8 can inhibit ferroptosis through modification of GPX4 and SLC7A11.^[^ ^23^, ^24^, ^25^ ^]^ However, our study demonstrated that ZDHHC20 and ZDHHC8 were not up‐regulated in C4‐2 cells after enzalutamide treatment (Figure 1J), indicating novel targets for enhancing the sensitivity of enzalutamide in CRPC. It is noteworthy that the development of anti‐tumor drugs related to the iron death pathway mainly focuses on GPX4, which is responsible for promoting lipid peroxide clearance.^[^ ^46^ ^]^

However, there is less agents targeting the lipid peroxide production, such as restoring the activity of ACSL4. ACSL4 is an enzyme critical for lipid metabolism that catalyzes the esterification of coenzyme A (CoA) into specific polyunsaturated fatty acids (PUFAs).^[^ ^57^, ^58^ ^]^ Among all ACSL family members, ACSL4 is the most potent promoter of ferroptosis.^[^ ^59^, ^60^ ^]^ ACSL4 mediates the sensitivity of RB1‐deficient prostate cancer cells to ferroptosis (^[^ ^61^ ^]^ b), and the downregulation of ACSL4 expression alleviates lipid oxidative stress,^[^ ^62^, ^63^, ^64^ ^]^ enabling CRPC cells to evade ferroptosis.^[^ ^65^ ^]^ Considering the crucial role of ACSL4 in cancer progression, diverse post‐translational modifications of ACSL4 have been explored. CDK1 phosphorylates ACSL4 at S447, triggering UBR5‐mediated polyubiquitination at K388/K498/K690 and subsequent proteasomal degradation.^[^ ^66^ ^]^ Conversely, TRIM28 stabilizes ACSL4 via SUMO3 conjugation at K532, which competitively inhibits K63‐linked ubiquitination and OPTN‐dependent autophagic clearance.^[^ ^60^ ^]^ Additionally, PKCβII‐mediated T328 phosphorylation activates ACSL4 enzymatic activity while establishing a lipid peroxidation‐PKCβII‐ACSL4 feedforward loop that amplifies ferroptosis signaling (^[^ ^67^ ^]^ b). Despite transcription regulation of ACSL4 by miR‐23a‐3p^[^ ^68^ ^]^ and YAP^[^ ^69^ ^]^ has been reported, investigations into its post‐translational modifications remain in nascent stages. During our investigation of enzalutamide resistance in prostate cancer, we revealed a novel crosstalk mechanism between palmitoylation and ferroptosis that involves lipid peroxide production. We discovered that USP19 inhibits the ubiquitin–proteasome pathway‐mediated degradation of ACSL4 and that ZDHHC2 enhances ACSL4 degradation by palmitoylating USP19, ultimately suppressing ferroptosis.

Notably, the crosstalk between ubiquitination and palmitoylation has been increasingly reported. For example, the palmitoylation and ubiquitination of MAVS jointly regulate antitumor immunity^[^ ^70^ ^]^; the palmitoylation‐mediated ubiquitination of PHF2 remodels lipid metabolism in hepatocellular carcinoma^[^ ^71^ ^]^; and the palmitoylation of FASN competes with ubiquitination to promote hepatocellular carcinoma progression.^[^ ^72^ ^]^ Our findings further elucidate the crosstalk between palmitoylation and ubiquitination in prostate cancer.

This study has several limitations. First, our metabolomics analysis did not detect palmitic acid in tumor samples, and the role of palmitoylation was inferred based on SM metabolic processes. Further investigation into the role of palmitic acid in prostate cancer is warranted. Second, we focused solely on the mechanism of ZDHHC2, leaving the roles of other ZDHHCs in CRPC unexplored. Third, while we investigated the ferroptosis‐mediated mechanism by which ZDHHC2 sustains the survival of CRPC cells after enzalutamide treatment, other potential processes were not examined in depth. Finally, the inhibitor TTZ1 we synthesized is just one of many possible ZDHHC2 inhibitors, and the efficacy of others remains unknown.

In summary, our study revealed a novel mechanism by which ZDHHC2 mediated S‐palmitoylation modulates ferroptosis and castration sensitivity in prostate cancer via controlling lipid peroxide production. We demonstrated that enzalutamide induces the abnormal upregulation of ZDHHC2 involving the FOXA1/CXXC5/TET2 complex decreased the sensitivity of enzalutamide to CRPC in a palmitoyltransferase‐dependent manner. Then, we found that ZDHHC2 decreases the lipid peroxide production and suppresses ferroptosis through mediating the S‐palmitoylation of USP19 and destabilizing ACSL4 in CRPC cells. Considering fewer chemical agents targeting lipid peroxide production‐related proteins, such as ACSL4, compared to those targeting lipid peroxide clearance‐related proteins, such as GPX4. We synthesize specifically inhibitors of ZDHHC2‐TTZ1. Therefore, our results reveal that ZDHHC2 is an ideal target for overcoming the resistance of CRPC to enzalutamide, and highlight the potential application of ZDHHC2 inhibitor in anticancer therapy.

## 4. Star*Methods

The key resources are provided in **Table** **1**.

**Table 1:** Key resources.

Reagent or Resource	Source	Identifier
Antibodies		
Rabbit polyclonal anti‐ZDHHC2	Affinity	Cat #DF4688 RRIDs: AB_2837039
Rabbit polyclonal anti‐HA	Proteintech	Cat# 51064‐2‐AP RRIDs: AB_11042321
Rabbit polyclonal anti‐Flag	Proteintech	Cat# 20543‐1‐AP RRIDs: AB_11232216
Mouse monoclonal anti‐Flag	Proteintech	Cat# 66008‐4‐Ig RRIDs: AB_2918475
Rabbit polyclonal anti‐Beta Actin	Proteintech	Cat# 20536‐1‐AP RRIDs: AB_10700003
Rabbit polyclonal anti‐ACSL4	Proteintech	Cat# 22401‐1‐AP RRIDs: AB_2832995
Rabbit polyclonal anti‐USP19	Proteintech	Cat# 25768‐1‐AP RRIDs: AB_2713918
Rabbit polyclonal anti‐AR	Proteintech	Cat# 22089‐1‐AP RRIDs: AB_11182176
Rabbit polyclonal anti‐FOXA1	Abcam	Cat# ab23738 RRIDs: AB_2104842
Rabbit polyclonal anti‐H3K27ac	Abcam	Cat# ab4729 RRIDs: AB_2118291
Rabbit polyclonal anti‐CXXC5	Proteintech	Cat# 16513‐1‐AP RRIDs: AB_2878269
Rabbit polyclonal anti‐TET2	Abcam	Cat# ab94580 RRIDs: AB_10887588
Rabbit polyclonal anti‐ID1	Proteintech	Cat# 18475‐1‐AP RRIDs: AB_2248812
Mouse monoclonal anti‐PFN2	Proteintech	Cat# 60094‐2‐Ig RRIDs: AB_2163215
RABBIT IgG	Abcam	Cat# ab171870 RRIDs: AB_2687657
Bacterial and virus strains		
*E. coli* BL21	Thermo Fisher	Cat# C600003
Chemicals, peptides, and recombinant proteins		
Erastin	Selleck	Cat# S7242
2‐BP	Selleck	Cat# E0120
biotin‐HPDP	Merck	Cat#SML3797
streptavidin	YEASEN	Cat# 47503ES03
Click‐iT palmitic acid azide	Thermo Fisher Scientific	Cat# C10265
Click‐iT Protein Reaction Buffer Kit	Thermo Fisher Scientific	Cat# C10276
MG132	MCE	Cat# HY‐13259
Cycloheximide	MCE	Cat# HY‐12320
RIPA lysis buffer	Servicebio	Cat#G2002‐100ML
protease inhibitor	Beyotime	Cat#P1005
phosphatase inhibitor	Beyotime	Cat#P1045
enhanced chemiluminescence luminescence solution	Thermo Fisher Scientific	Cat#34577
glutathione‐agarose beads	Thermo Fisher Scientific	Cat#16100
Coomassie Brilliant Blue staining	Servicebio	Cat#G2059
TRIzol reagent	Accurate Biology	Cat#AG21102
Critical commercial assays		
reverse transcription kit	Accurate Biology	Cat#AG11728
Evo M‐MLV one‐step RT‐qPCR kit	Accurate Biology	Cat#AG11732
Commercial chromatin extraction kit	Abcam	Cat#ab117152
ChIP kit Magnetic‐One Step	Abcam	Cat#ab156907
Prostate cancer tissue microarray chip	Zhongke Guanghua	Cat#D097LV01
ROS detection kit	Beyotime	Cat#S0033S
Cellular MDA assay kit	Beyotime	Cat#S0131S
Micro‐reduced glutathione assay kit	Solarbio	Cat#BC1175
CCK‐8 kit	Beyotime	Cat#C0037
Experimental models: Cell lines		
Human: C4‐2	Yuchicell	Cat#SC1945
Human: 22Rv1	Yuchicell	Cat#SC0129
Human: LNCaP	Yuchicell	Cat#SC0127
Oligonucleotides		
siRNAs, shRNAs and sgRNAs, see Table S2 (Supporting Information)	Supplementary data	N/A
Primers for RT‐qPCR, see Table S3 (Supporting Information)	Supplementary data	N/A
Primers for ChIP‐qPCR, see Table S4 (Supporting Information)	Supplementary data	N/A
Recombinant DNA		
HA‐FOXA1	Gene chem	N/A
Flag‐CXXC5 WT	Gene chem	N/A
Flag‐CXXC5 MT	Gene chem	N/A
Flag‐TET2 WT	Gene chem	N/A
Flag‐TET2 H1881R	Gene chem	N/A
Flag‐ZDHHC2 MT	Gene chem	N/A
Flag‐ZDHHC2 C129A	Gene chem	N/A
HA‐USP19 WT	Gene chem	N/A
HA‐USP19 C152S	Gene chem	N/A
HA‐Ub	Gene chem	N/A
Software and algorithms		
ImageJ	NIH	N/A
GraphPad Prism 7.0	GraphPad, lnc	N/A
R Studio v1.2.5042	Posit	N/A

## 4 Experimental Model and Subject Details

### Cell Line and Cell Culture

This study utilized the human prostate cancer cell lines C4‐2, 22Rv1, and LNCaP. All cell lines were obtained from Procell Life Science & Technology Co., Ltd (China) and underwent rigorous short tandem repeat (STR) profiling to ensure authenticity. The cells were cultivated in a humidified incubator at 37 °C with 5% CO2. C4‐2, 22Rv1, and LNCaP cell lines were cultured in Roswell Park Memorial Institute (RPMI) 1640 medium supplemented with 10% fetal bovine serum (FBS) and 1% penicillin‐streptomycin. All media and serum were sourced from Gibco (DMEM: #11320033, RPMI 1640: #11875093, serum: #A5256701, USA), while penicillin–streptomycin was obtained from Thermo Fisher Scientific (#15140122, USA).

### Patient Specimens

Patients with CRPC who had been treated with enzalutamide were enrolled, approved by the Medical Committee of the Second Xiangya Hospital of Central South University (No. LYEC2024‐0076). Written informed consent was obtained from all patients for the use of their clinical samples and data in this study. During surgery, prostate cancer tissues were collected from all patients. Pathology experts conducted a double‐blind evaluation to confirm the diagnosis. In total, five CRPC patients sensitive to enzalutamide and five resistant patients were collected. Patient information is summarized in Table S1 (Supporting Information).

### Mice Xenograft Model

All animal procedures were approved by the Ethics Committee of the Second Xiangya Hospital of Central South University (No. 20241019). Six‐week‐old male nude mice were obtained from Vital River. The mice were housed in a specific pathogen‐free (SPF) animal facility. All animals were randomly assigned to groups. For cell line‐derived xenograft (CDX), five million (5×10⁶) cells were injected subcutaneously into each mouse. For patient‐derived xenografts, tumor lines were grafted in NSG mice as previously described.^[^ ^73^ ^]^ At designated time points, animals were euthanized, and tumors were excised, weighed, and photographed. The animal experiment complied with the NIH Guide for the Care and Use of Laboratory Animals (8th ed., 2011).

### Genetically Engineered Mice

Genetically engineered mice were designed by Shanghai Model Organisms Center, Inc (SMOC). *Zdhhc2* knockout mice were generated using CRISPR/Cas9 gene editing technology. The targeting region for knockout was selected as exons 2–6 of the *Zdhhc2*‐201 transcript based on the gene structure. Specifically, guide RNA sequences were designed for intron 1 and intron 6 of the *Zdhhc2* gene, with high‐scoring candidates selected using crispor.tefor.net. The guide RNAs and Cas9 mRNA were microinjected into fertilized eggs. Positive F0 mice were screened by PCR and sequencing, and then bred with wild‐type mice to produce F1 offspring. Genotyping of F1 mice was confirmed by PCR and sequencing. Finally, 6‐8‐week‐old positive F1 mice were used for subsequent experiments.

### Spontaneous Prostate Cancer Model

A plasmid mixture containing pT3‐AKT (5 µg), pT3‐MYC (5 µg), and pCAG‐SB100 (1 µg) was prepared in Ringer's solution (10 µL per mouse). Mice were anesthetized via intraperitoneal injection of tribromoethanol. After abdominal disinfection with ethanol, surgery was performed to expose the prostate. The plasmid mixture was injected into the prostatic with an insulin syringe. After 2 weeks, the animals were euthanized, and their prostates were collected.

### Transient Transfection

Cells were cultured in plates and starved for 6 h in Opti‐MEM (#31985070, Gibco, USA). Subsequently, plasmid or siRNA was combined with 250 µL of Opti‐MEM. Lipofectamine 2000 (#11668019, Thermo Fisher Scientific, USA) was combined with 250 µL of Opti‐MEM. After 5‐min incubation, the plasmid or siRNA and Lipofectamine mixture was then mixed and incubated for an additional 20 min. This transfection mixture was added to 3500 µL of Opti‐MEM, and cells were transfected for 7 h. After transfection, Opti‐MEM was replaced with complete medium. The sequences of all small interfering RNAs (siRNAs) and short hairpin RNAs (shRNAs) were listed in Table S2 (Supporting Information).

### Western Blot Analysis

Cells were lysed on ice for 30 min using RIPA lysis buffer (#G2002‐100ML, Servicebio, China), supplemented with 1% protease inhibitor (#P1005, Beyotime, China) and 1% phosphatase inhibitor (#P1045, Beyotime, China). Cell lysates were centrifuged at 12,000 rpm and 4 °C for 10 min, and the supernatant was collected to measure protein concentration with Micro BCA protein assay kit. After adding the loading buffer, the supernatant was boiled at 95 °C for 10 min. Proteins were separated by sodium dodecyl sulfate polyacrylamide gel electrophoresis (SDS‐PAGE) and transferred to polyvinylidene difluoride (PVDF) membranes. Following a 1‐h blocking step with skim milk at room temperature, the membranes were incubated with primary antibodies at 4 °C overnight. The second day, the membranes were incubated with secondary antibodies at room temperature for 1 h, and protein signals were developed using enhanced chemiluminescence (ECL) luminescence solution (#34577, Thermo Fisher Scientific, USA).

### Coimmunoprecipitation

Cell lysates were prepared in RIPA lysis buffer supplemented with protease and phosphatase inhibitors. After centrifugation, the supernatant was incubated overnight at 4 °C with specific antibodies and magnetic beads. Then the beads were washed six times to remove non‐specifically bound proteins and treated with the sample buffer and boiled. Finally, proteins were detected by Western blot.

### Glutathione S‐Transferase (GST) Pull‐Down Assay

To extract GST fusion proteins expressed in the BL21 strain (vector: pET‐GST), we first lysed the bacteria with muramidase and sonicated them. Subsequently, the GST fusion proteins were captured overnight at 4 °C with glutathione–agarose beads (#16100, Thermo Fisher Scientific, USA). After repeated washing, the captured proteins were mixed with cell lysate of the target protein (lysed on ice for 30 min in Western/IP lysis buffer) and incubated overnight at 4 °C. After multiple washings and boiling, the proteins were separated by SDS‐PAGE, and the gel was stained and imaged using Coomassie Brilliant Blue staining (#G2059, Servicebio, China) to provide a basis for subsequent analysis.

### Quantitative Real‐Time PCR (RT‐qPCR)

Total RNA was extracted with TRIzol reagent (#AG21102, Accurate Biotechnology, China). A NanoDrop2000 spectrophotometer (Thermo Fisher Scientific, USA) was used to quantify RNA. A reverse transcription kit (#AG11728, Accurate Biology, China) was used to reverse transcribe RNA into cDNA. The gene‐specific primers used in the experiment were synthesized by BGI (Beijing), and the sequences are listed in Table S3 (Supporting Information). Finally, the expression level of the target gene was quantitatively analyzed with Evo M‐MLV one‐step RT‐qPCR kit (#AG11732, Accurate Biology, China).

### Chromatin Immunoprecipitation (ChIP)‐qPCR Analysis

Commercial chromatin extraction kit (#ab117152, Abcam, USA) and ChIP kit Magnetic‐One Step (#ab156907, Abcam, USA) were used. The specific primer sequences used in ChIP‐qPCR are provided in Table S4 (Supporting Information).

### Immunohistochemistry

Prostate cancer tissue microarray chip (#D097LV01) from Zhongke Guanghua (Xi'an) Intelligent Biotechnology Co., Ltd. was used for immunohistochemistry (IHC) staining. We employed the IHC staining kit and corresponding antibodies from Bios Biological Technology Co., Ltd. to stain the tissue chip. Two experienced pathologists independently evaluated the IHC staining results. The staining intensity was categorized into three levels at 40× magnification: Grade 1 (weak): little staining; Grade 2 (moderate): moderate staining; Grade 3 (strong): strong staining.

### Intracellular Reactive Oxygen Species (ROS) Detection

For reactive oxygen species (ROS) detection, a ROS detection kit (#S0033S, Beyotime, China) and fluorescent probe DCFH‐DA were used. DCFH‐DA was diluted 1:1000 with serum‐free medium to a final concentration of 10 µm L^−1^. The cells were suspended in the diluted DCFH‐DA and incubated in a cell culture incubator at 37 °C for 20 min. After washing with PBS three times, the cells were resuspended in 200 µL PBS and analyzed by flow cytometry (Beckman Coulter, CA, USA).

### Lipid Peroxidation Detection

For the detection of lipid peroxidation, the BODIPY 581/591 C11 probe (Thermo Fisher Scientific) was used as reported before.^[^ ^61^ ^]^ Briefly, treated cells were seeded uniformly in six‐well plates and loaded with 10 µm BODIPY C11 at 37°C for 30 min. Cells were then harvested by trypsinization and resuspended in PBS. The cell suspension was transferred to a black‐walled 96‐well plate, and fluorescence signals were measured using a microplate reader. Lipid peroxidation was quantified by calculating the ratio of oxidized (Ex/Em = 485/535 nm) to reduced (Ex/Em = 560/591 nm) C11 fluorescence signals.

### MDA Detection

For MDA detection, a cellular MDA assay kit (#S0131S, Beyotime, China) was used employing the thiobarbituric acid method. And the MDA level was adjusted based on the cell protein concentration.

### Transmission Electron Microscopy (TEM)

The trypsin‐digested cells were terminated with complete culture solution and centrifugally collected. The cell particles were transferred into a clean 1.5 mL EP tube and centrifuged at 1000 rpm for 5–10 min. Discard the supernatant and retain the cell particles. One milliliter of pre‐cooled 2.5% glutaraldehyde fixing solution (#P1126, Solarbio, China) was slowly added to the cell granules, and the cells were gently dispersed with toothpicks. The mixture was set in the dark at room temperature for 3–5 min, then transferred to 4 °C for storage. Finally, the ultrathin sections were examined by transmission electron microscopy (TECNA I20, Philips, Eindhoven, Netherlands) to analyze the morphological changes of mitochondria.

### Cell Counting Kit‐8 (CCK‐8) Assay

We used CCK‐8 kit (#C0037, Beyotime, China) to perform CCK‐8 assay. Cells from different treatment groups were collected, thoroughly resuspended in culture medium, and counted. The cell suspension was then diluted to ensure that each well of the 96‐well plate contained 5000 cells in 100 µL (adjusted based on cell viability). Each group included at least three replicates. Wells containing only culture medium (without cells) were reserved as blank controls.CCK‐8 solution was added to each well and incubated for 1 h. The absorbance of each well was measured at a wavelength of 450 nm using a microplate reader to assess cell viability.

### Proximity Ligation Assay (PLA)

Cells were seeded on sterile coverslips (#FCP126, Beyotime, China) in 12‐well plates and cultured in an incubator. After 36 h, the culture medium was removed, the cells were washed with PBS, and fixed and permeabilized. The cells were blocked with Duolink blocking solution to reduce nonspecific binding. Next, antibodies were incubated at 4 °C overnight. Subsequently, cells were washed twice with PBS containing 5% BSA and incubated in PLA probe mixture for 1 h. Ligation reaction and amplification reaction were performed in sequence according to the instructions of Duolink in situ detection kit (Sigma–Aldrich, USA). Finally, cells were rinsed with Duolink In Situ Wash Buffer, mounted, and imaged.

### Acyl‐Biotinyl Exchange (ABE) Assay

To exhaustively block free thiols, 20 mmol L^−1^ of methyl methanethiosulfonate (Merck) and 1 mmol L^−1^ PMSF (Beyotime, China) were used to incubate with cell lysates at 50 °C for 30 min. Proteins were precipitated with acetone, and then resuspended in 1 mol L^−1^ hydroxylamine pH 7.4 (Merck) to promote depalmitoylation. After that, the proteins were incubated with 0.2 mmol L^−1^ biotin‐HPDP (#SML3797, Merck) for 1 h at room temperature. Biotinylated proteins were purified with Streptavidin (Yeasen) and analyzed by immunoblotting.

### Click‐iT Pulldown

Click chemistry and streptavidin pulldown were performed for click‐iT identification of palmitoylation.^[^ ^74^ ^]^ Cells were incubated with 100 µmol L^−1^ of Click‐iT palmitic acid‐azide (Thermo Fisher Scientific) for 6 h, and then lysed for protein extraction. A Click‐iT Protein Reaction Buffer Kit (# C10276; Thermo Fisher Scientific) was used to catalyze the reaction between protein samples and biotin–alkyne. Streptavidin (Yeasen, China) was used to precipitate the biotin alkyne–azide–palmitic‐protein complex. For immunoblotting analysis, the bound proteins were eluted by boiling with SDS‐PAGE sample buffer for 10 min at 95 °C.

### CRISPR/Cas9 Technique

sgRNAs were designed through https://www.synthego.com. Then sgRNAs were cloned into the lentiCRISPR v2 vector (Addgene, #52961). The sgRNAs sequence is shown in Table S2 (Supporting Information).

### Non‐Targeted Metabolomics and Non‐Targeted Lipidomics Analysis

Non‐targeted metabolomics and non‐targeted lipidomics were analyzed by Shanghai BioProfile Biotechnology (China). In metabolomics analysis, we used a methanol/acetonitrile/water mixture to extract metabolites from cells and performed sample preparation through steps such as sonication and centrifugation. Subsequently, the extracts were analyzed using liquid chromatography‐mass spectrometry. In lipidomics analysis, we used methyl tert‐butyl ether (MTBE) to extract lipids from cells and enriched lipids through steps such as phase separation and centrifugation. Finally, ultra‐high performance liquid chromatography‐tandem mass spectrometry was used to analyze lipids, and the obtained data were preprocessed and statistically analyzed.

### Proteomics Analysis

For proteomics, the prepared tissue samples were further analyzed by Shanghai Bioproffle Technology Co., Ltd. Cell samples were lysed with 200 µL of lysis buffer (4% SDS, 100 mm DTT, 150 mm Tris‐HCl pH 8.0). The samples were boiled and further sonicated. Unlysed cell debris was removed by centrifugation at 16 000 × g for 15 min. The supernatant was collected and quantified by BCA. Subsequently, peptide fragment preparation, LC‐MS analysis, database searching and analysis, and bioinformatics analysis were performed. For MS data acquisition, timsTOF Pro2 (Bruker) was operated in PASEF mode. The full scan was recorded from 100 to 1700 m/z, and the mobility (1/K0) dimension spanned from 0.6 to 1.6 Vs cm^−2^. The MS method was set as follows: ramp time 100 ms, accumulation time 2.0 ms, lock duty cycle 100%, capillary voltage 1700V, drying gas 3 l/min, and drying temperature 180°C.

### Palmitoylation Proteomics Analysis

Palmitoylation proteomics was performed by Shanghai Bioproffle Technology Co., Ltd. Detailed information was shown in Supporting Information.

### Quantification and Statistical Analysis

The experimental data were statistically analyzed with GraphPad Prism 7.0. Statistical significance was determined by unpaired Student's t test (when comparing two experimental groups), one‐way and two‐way ANOVA tests as appropriate (when comparing more than two experimental groups). All statistical results are expressed as mean ± standard deviation, and *, **, *** indicate differences of *p* < 0.05, *p* < 0.01, and *p* < 0.001, respectively.

### Ethics Approval and Consent to Participate

It is confirmed that all methods were performed in accordance with the relevant guidelines and regulations. All animal procedures were approved by the Ethics Committee of the Second Xiangya Hospital of Central South University (No. 20241019). All animal experiments were performed following the U.S. Public Health Service Policy on Humane Care and Use of Laboratory Animals (2015 reprint). The collection of clinical samples was approved by the Medical Committee of the Second Xiangya Hospital of Central South University (No. LYEC2024‐0076). Informed consent from all patients for the use of their specimens was obtained. The study was conducted in accordance with the principles of the Declaration of Helsinki (WMA).

## Author's Contributions

S.S., W.L., and Y.H. contributed equally to this work. S.S., W.L., and Y.H. developed the methodology. R.Z. performed formal analysis. X.J. conceptualized the study. L.Z., L.Y., Y.L., Y.W., H.H., and X.J. performed the investigation. X.J., H.H., and X.J. performed project administration.

## Conflict of Interest

The authors declare no conflict of interest.

## Supporting information



Supporting Information

## Data Availability

The data that support the findings of this study are available from the corresponding author upon reasonable request.
