# Supplementary material for: ZDHHC2‐Dependent Palmitoylation Dictates Ferroptosis and Castration Sensitivity in Prostate Cancer via Controlling ACSL4 Degradation and Lipid Peroxidation
Source: Adv Sci (Weinh). 2025 Oct 23;13(1):e14077. doi: 10.1002/advs.202514077 (PMC12767118; doi:10.1002/advs.202514077)
Supplement: Supplementary file 1 — Supporting Information [file ADVS-13-e14077-s001.docx]

**Palmitoylation proteomics analysis**

Tissue samples were weighed, then washed with ice-cold PBS 2 times and ground into powder using liquid nitrogen. Approximately 50 mg of powder were resuspended in 200 µl lysis buffer (4% SDS, 150 mM Tris-HCl pH 8.0). Cell samples were dissolved with 200 µl lysis buffer (4% SDS, 150 mM Tris-HCl pH 8.0). All samples were sonicated for 2 min on cold water bath. Then proteins were extracted by centrifugation and determined the concentration by BCA method. Protein (3 mg) from each sample was precipitated with pre-cooled acetone and the free cysteines of proteins were blocked by mixing for 12 h at 4 °C in 5 ml total PBS containing 0.5% SDS, 1% Triton X-100 (Sigma-Aldrich), protease inhibitors (Thermo Scientific), 5 mM EDTA and 25 mM N-ethylmaleimide (NEM, Thermo Scientific). The samples were precipitated using chloroform/methanol to remove excess NEM. Proteins were resuspended in 1.5 ml of resuspension buffer (4% SDS, protease inhibitors (Thermo Scientific), 5 mM EDTA in PBS pH 7.4), and mixed with 3 ml of 1 M hydroxylamine (pH 7.4 with NaOH, Thermo Scientific), and 500 µl of 4 mM Biotin-HPDP (Thermo Scientific) in DMSO, and then incubated at 25 °C for 2 h with gentle mixing. Proteins were precipitated again and resuspended in 6 M urea and then diluted six-fold with 50mM ammonium bicarbonate, and then digested with trypsin at the enzyme-to-protein ratio of 1:50 into the sample and incubated at 37℃ for 20 h. The tryptic digested peptides were first desalted with C18 spin column (Thermo Scientific). The dried peptides were resuspended with 200 ul loading buffer (0.2% SDS, 0.2% Triton X-100 and 500 mM NaCl) and mixed with 100 µl of high-capacity streptavidin beads (Thermo Scientific) for 2 h at room temperature. Beads were washed three times with 5 ml PBS containing 0.2% SDS, 0.2% Triton X-100 and 500 mM NaCl and then collected after wash with 1 ml PBS twice. The beads were then incubated with 0.2 ml elution buffer (50 mM NH4HCO3, 10 mM TCEP) for 2 h at room temperature. The eluted peptides were collected and mixed with 50 mM iodoacetamide to block reduced cysteine residues, which indicated the palmitoylation sites. Finally, the peptides were desalted with C18 Stage Tips and prepared for further LC-MS/MS analysis. Liquid chromatography-mass spectrometry (nanoLCMS/MS) was performed on a Q Exactive HF-X (Thermo Scientific) coupled with Easy nLC 1200 system for chromatographic separation. Peptides were loaded onto a C18 column (20 cm long, 75 μm ID, 2 μm, Dr. Maisch GmbH, Ammerbuch, Germany)) in buffer A (2% ACN and 0.1% FA) and separated with a linear gradient of buffer B (90% ACN and 0.1% FA) at a flow rate of 300 nl/min over 120 min. The linear gradient was set as follows: 0–2 min, linear gradient from 2 to 5% buffer B; 2–82 min, linear gradient from 5 to 20% buffer B; 82–100 min, linear gradient from 20 to 35% buffer B; 100–112 min, linear gradient from 35 to 90% buffer B; 112–120 min, buffer B maintained at 90%. Mass spectrometry was performed on a nano electrospray ion source (nESI). The spry voltage was set as 2.1 kv. For MS data acquisition, the full MS scans were surveyed from m/z 350 to m/z 1800 at a resolution of 60,000 at m/z 200 with an AGC target values of 3e6 and a maximum injection time 50 ms. A lock mass of 445.120025 Da was used as internal standard for mass calibration. Then data-dependent top 20 MS/MS scans were applied by higher-energy collision dissociation (HCD) with normalized energy 28 at a resolution of 15,000 at m/z 200 with an AGC target values of 1e5 and a maximum injection time 50 ms. The isolation window was set to 1.6 Th and dynamic exclusion duration was 30 s.

**
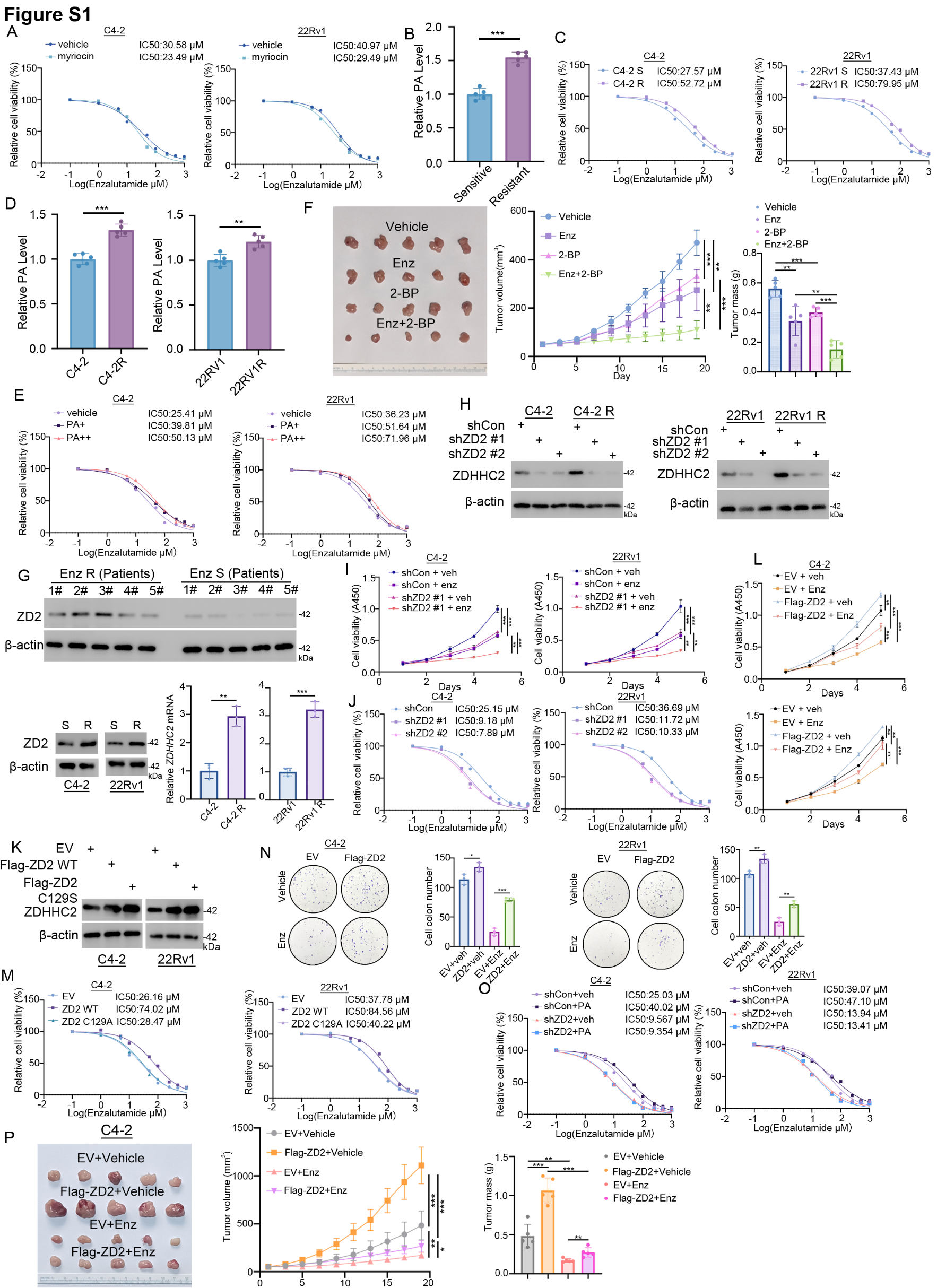
**

**Figure S1.** **Palmitoylation contributes to the survival of CRPC cells after enzalutamide treatment**

A, C4-2 or 22Rv1 cells were treated with myriocin and a serial dose of enzalutamide for 24 h, followed by CCK-8 assays. B, palmitic acid level was detected in enzalutamide-sensitive and resistant patient prostate samples. Data were expressed as mean ± SD, five replicates. ***, p < 0.001. C, C4-2 and 22RV1 cells were treated with enzalutamide or vehicle (DMSO) to establish enzalutamide-resistant cell lines. After treatment, resistant cells were cultured in 96-well plates and treated with different concentrations of enzalutamide for 24 hours. The corresponding IC50 values are indicated. D, palmitic acid level was detected in enzalutamide-sensitive and resistant cell lines. Data were expressed as mean ± SD, five replicates. **, p < 0.01; ***, p < 0.001. E, C4-2 or 22Rv1 cells were treated with palmitic acid and a serial dose of enzalutamide for 24 h, followed by CCK-8 assays. F, patient-derived prostate cancer cells were subcutaneously injected into nude mice, which were then treated with or without enzalutamide (10 mg/Kg) in combination with 2-BP (5 mg/Kg). Representative tumor images are shown. Data were expressed as mean ± SD, five replicates. **, p < 0.01; ***, p < 0.001. G, protein and mRNA analysis of ZDHHC2 protein expression in enzalutamide-resistant and -sensitive patient prostate cancer samples and C4-2 and 22Rv1 cells. H, 22Rv1 and C4-2 cells were infected with indicated shRNAs for 72 hours (puromycin-selected), western blot was performed for verification. I, indicated constructs were transfected into C4-2 and 22Rv1 cells for 72 hours. Cells were treated with or without enzalutamide (10 μM) for 24 hours and cultured in 96-well plates for CCK-8 analysis. Data were expressed as mean ± SD, three replicates. **, p < 0.01; ***, p < 0.001. J, indicated constructs were transfected into cells for 72 hours. Cells were cultured in 96-well plates and then treated with different concentrations of enzalutamide for 24 hours. The corresponding IC50 values are indicated. K, cells infected with indicated plasmids and western blot was performed for verification. L, indicated constructs were transfected into cells for 24 hours and cells were cultured with or without enzalutamide (10 μM) for CCK-8 analysis. Data were expressed as mean ± SD, three replicates. **, p < 0.01; ***, p < 0.001. M, indicated constructs were transfected into cells for 24 hours. Cells were cultured in 96-well plates and then treated with different concentrations of enzalutamide for 24 hours. The corresponding IC50 values are indicated. N, indicated constructs were transfected into cells for 24 hours and then the cells were treated with or without enzalutamide (10 μM) for 24 hours. Cells were cultured in 6-well plates for colony formation assays. Data were expressed as mean ± SD, three replicates. *, p < 0.05; **, p < 0.01; ***, p < 0.001. O, indicated constructs were transfected into cells for 72 hours. After palmitic acid or vehicle treatment, cells were cultured in 96-well plates and then treated with different concentrations of enzalutamide for 24 hours. The corresponding IC50 values are indicated. P, C4-2 cells infected with indicated plasmids and subcutaneously injected into nude mice, which were then treated with or without enzalutamide (10 mg/Kg). Tumor growth curve and mass are shown. Data represent mean ± SD (n=5). *, p < 0.05; **, p < 0.01; ***, p < 0.001.

**
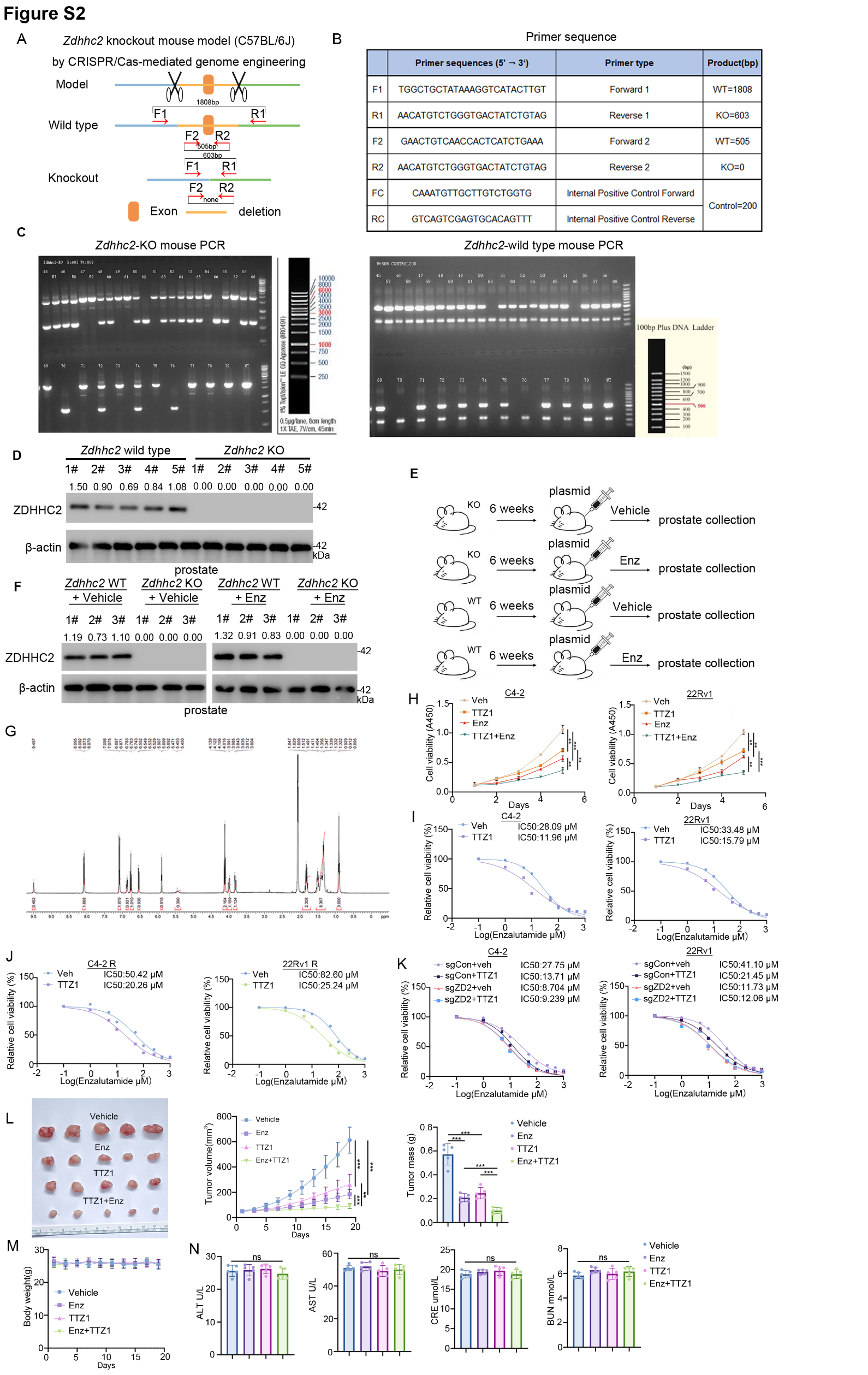
**

**Figure S2. ZDHHC2 contributes to the survival of CRPC cells after enzalutamide treatment**

A, schematic representation of the Zdhhc2 knockout mouse model generated by CRISPR/Cas-mediated genome engineering. The target region and primer binding sites (F1, R1, F2, R2) are indicated for both wild-type and knockout alleles. B, primer sequences used for genotyping and internal positive control PCR. Expected product sizes for wild-type (WT) and knockout (KO) alleles are listed. C, representative gel electrophoresis images of PCR products from genomic DNA of *Zdhhc2* knockout mice (left panel) and wild-type mice (right panel) using the primers described in (B). D, western blot analysis of ZDHHC2 protein expression in prostate tissue lysates from wild-type and Zdhhc2 knockout mice (n=5 per group). E, schematic diagram illustrating the experimental design for in vivo enzalutamide treatment. F, western blot analysis of ZDHHC2 protein expression in prostate tissue lysates from wild-type and *Zdhhc2* knockout mice treated with vehicle or enzalutamide (n=3 per group). G and H, enzalutamide sensitive and resistant C4-2 or 22Rv1 cells were treated with TTZ1 (10 μM) and a serial dose of enzalutamide for 24 h, followed by CCK-8 assays. I-J, cells were treated with or without TTZ1 (10 μM) for 24 hours and then were treated with different concentrations of enzalutamide for 24 hours. The corresponding IC50 values are indicated. K, endogenous ZDHHC2 was knocked out in C4-2 and 22Rv1 cells with CRISPR/Cas9. The ZDHHC2-knockout and control cells were treated with vehicle or TTZ1 (10 μM) for 24 hours and then were treated with different concentrations of enzalutamide for 24 hours. The corresponding IC50 values are indicated. L-N, patient-derived prostate cancer cells were subcutaneously injected into nude mice, and treated with or without enzalutamide (10 mg/Kg) in combination with TTZ1 (10 mg/Kg). Representative tumor images are shown. Body weight, liver function and kidney function of the mice were detected. ns, not significant; **, p < 0.01; ***‌, p < 0.001.

**
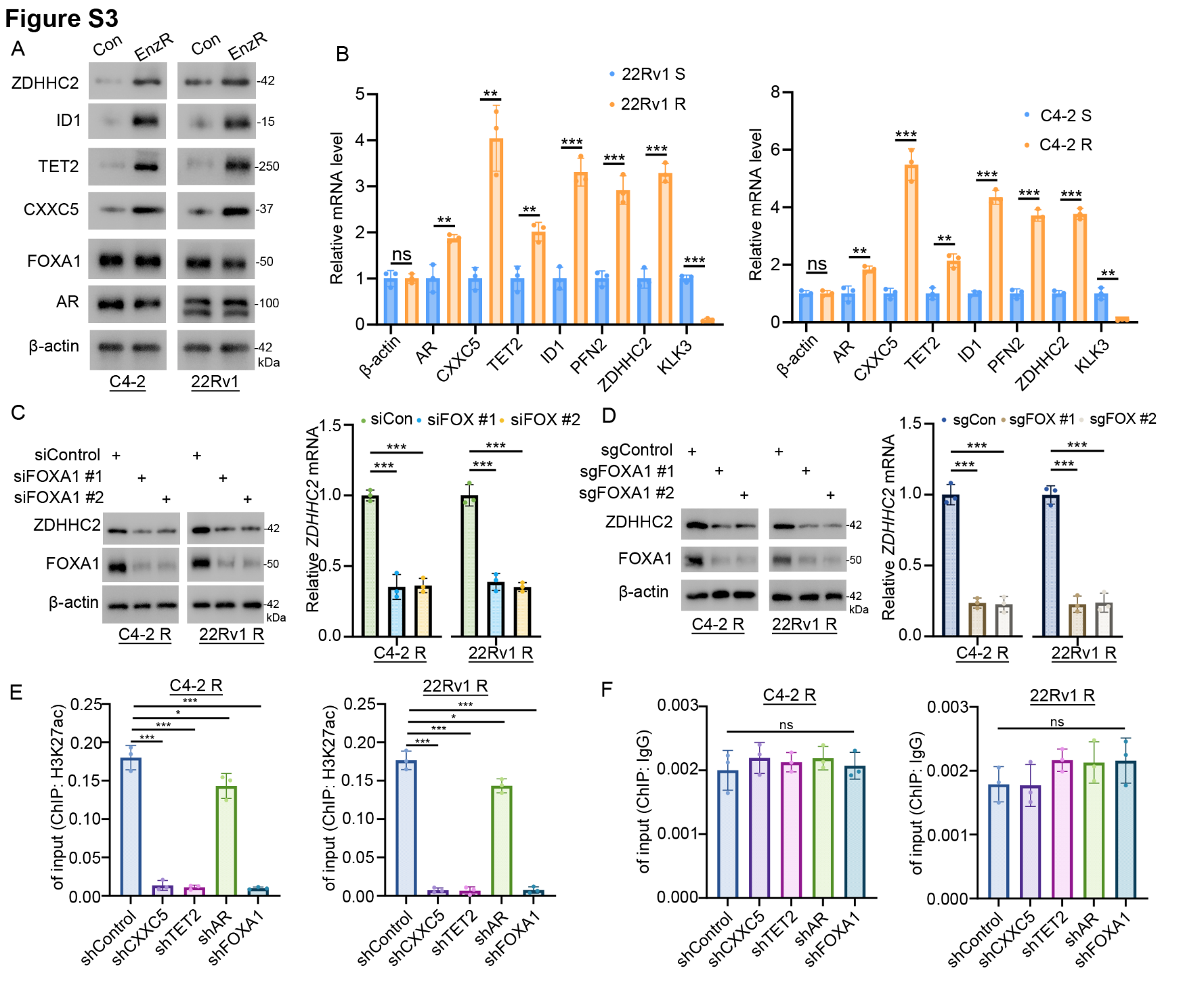
**

**Figure S3. ZDHHC2 is transcriptionally upregulated by the FOXA1/CXXC5/TET2 complex in ENZ-resistant CRPC cells**

‌ A-B, enzalutamide-resistant C4-2 and 22Rv1 cells were analyzed by Western blot (‌A‌) and RT-qPCR(‌B‌). Data represent mean ± SD (n=3). ns, not significant; **, p < 0.01; ***‌, p < 0.001. C, enzalutamide-resistant C4-2 and 22Rv1 cells were transfected with FOXA1 siRNA for 48 h. Cells were harvested for Western blot and RT-qPCR analysis of ZDHHC2 expression. Data represent mean ± SD (n=3). ‌***‌, p < 0.001. D, enzalutamide-resistant C4-2 and 22Rv1 cells were transfected with FOXA1 sgRNA (in combination with Cas9) for 72 h. Cells were harvested for Western blot and RT-qPCR analysis of ZDHHC2 expression. Data represent mean ± SD (n=3). ‌***‌, p < 0.001. E, ChIP-qPCR analysis of H3K27ac enrichment at genomic loci of ZDHHC2 in enzalutamide-resistant C4-2 and 22Rv1 cells transfected with control, CXXC5-, TET2-, AR-, or FOXA1-specific shRNAs. Data represent mean ± SD (n=3). *, P < 0.05; ***‌, P < 0.001. F, ChIP-qPCR analysis of nonspecific background signals using normal IgG immunoprecipitation at genomic loci of ZDHHC2 in enzalutamide-resistant C4-2 and 22Rv1 cells transfected with control, CXXC5-, TET2-, AR-, or FOXA1-specific shRNAs. Data represent mean ± SD (n=3). ns, not significant.


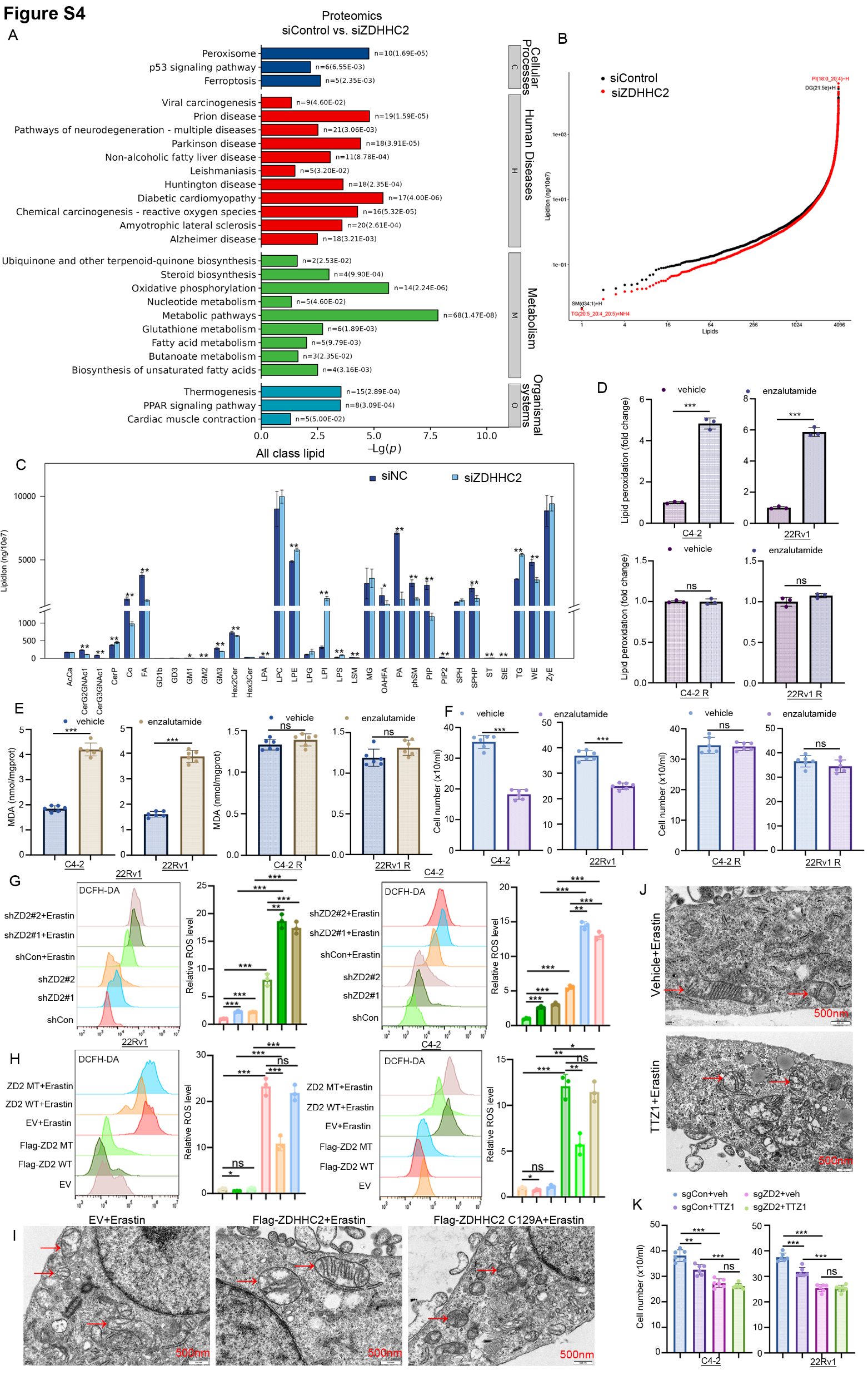


**Figure S4. ZDHHC2 promotes enzalutamide resistance by inhibiting lipid peroxide production and ferroptosis**

‌A,‌ C4-2 cells with or without ZDHHC2 knockdown were harvested for proteomic analysis, followed by pathway enrichment analysis of differentially expressed proteins‌. ‌B and C,‌ C4-2 cells with or without ZDHHC2 knockdown were subjected to lipid metabolomics analysis to quantify major lipid levels and composition‌. D-F, enzalutamide-sensitive and -resistance C4-2 and 22Rv1 cell lines were treated with enzalutamide, followed by analysis of lipid peroxidation (D), malondialdehyde (MDA) levels (E), and cell counting (F). Data represent mean ± SD from three or six replicates. ns, not significant; ***‌, p < 0.001. G, 22Rv1 and C4-2 cells were infected with indicated shRNAs for 72 h. After puromycin selection, cells were treated with or without Erastin (10 μM) for 24 h, followed by analysis of lipid ROS. Data represent mean ± SD from three replicates. ns, not significant; *, p < 0.05; **, p < 0.01; ***‌, p < 0.001. H, 22Rv1 and C4-2 cells were transfected with indicated plasmids for 24 h, then treated with or without Erastin (10 μM) for 24 h and analyzed for lipid ROS. Data represent mean ± SD from three replicates. ns, not significant; *, p < 0.05; **, p < 0.01; ***‌, p < 0.001. I,‌ 22Rv1 cells transfected with indicated plasmids for 24 hours were treated with Erastin (10 μM) for 24 hours, then processed for transmission electron microscopy (TEM) to assess mitochondrial ultrastructure‌. J,‌ 22Rv1 were treated with TTZ1 (10 μM) and Erastin for 24 hours and performed TEM for mitochondrial morphology. K, endogenous ZDHHC2 was knocked out in C4-2 and 22Rv1 cells with CRISPR/Cas9. The ZDHHC2-knockout and control cells were treated with vehicle or TTZ1 (10 μM) for 24 hours and analyzed for cell counting. Data represent mean ± SD from six replicates. ns, not significant; **, p < 0.01; ***, p < 0.001.

**
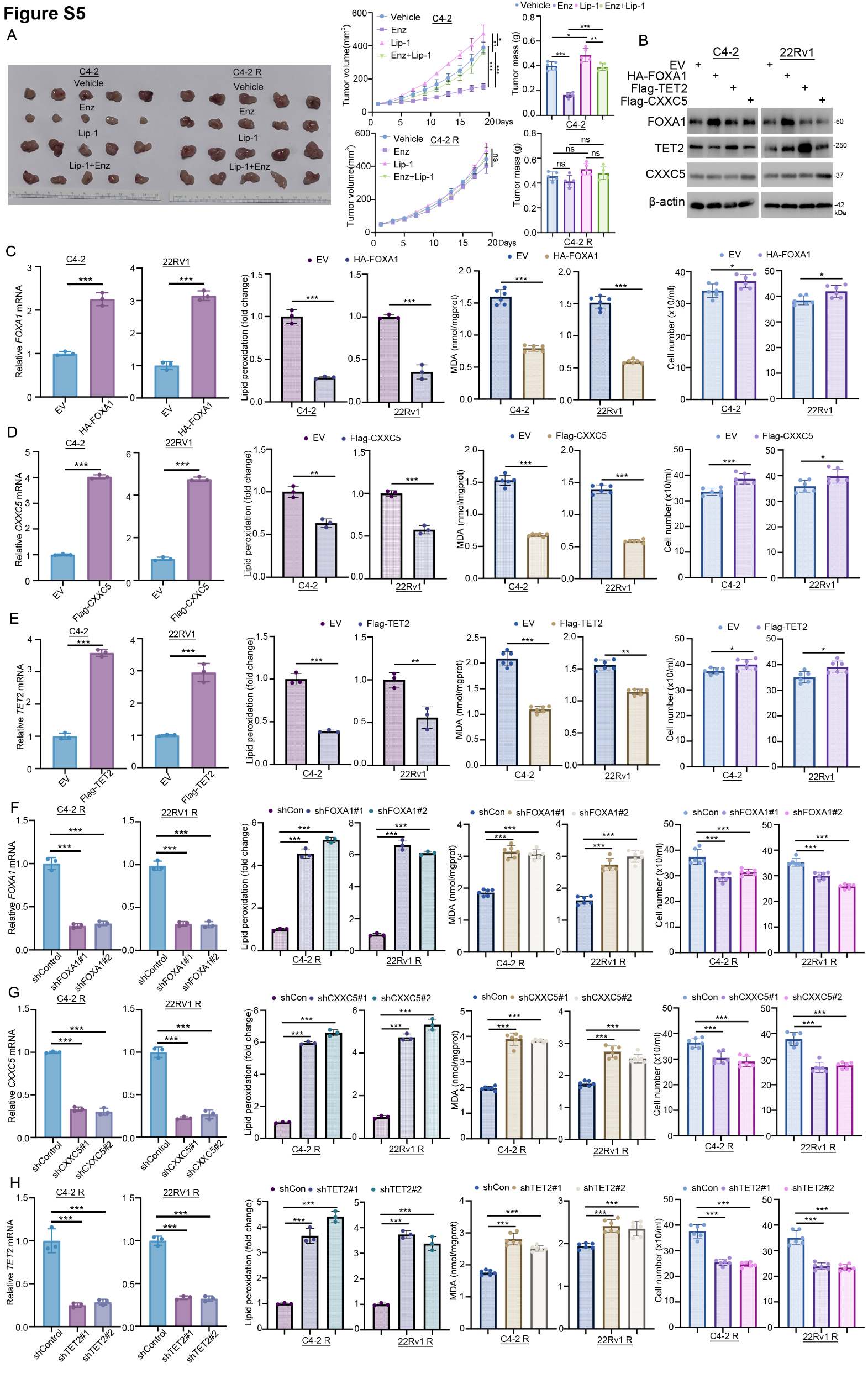
**

**Figure S5. The FOXA1/CXXC5/TET2 complex promotes enzalutamide resistance by ferroptosis inhibition**

‌A,‌ enzalutamide sensitive and resistant C4-2 cells were injected into nude mice, which were then treated with or without enzalutamide in combination with Lip-1. Tumor growth curve and mass are shown. Data represent mean ± SD (n=5). ns, not significant; *, p < 0.05; **, p < 0.01; ***, p < 0.001. B, cells infected with indicated plasmids and western blot was performed for verification. C-E, mRNA level, lipid peroxidation, MDA and cell counting of enzalutamide-sensitive C4-2 and 22Rv1 cell lines after overexpression of FOXA1 (C), CXXC5 (D) or TET2 (E). Data represent mean ± SD (n=3 or 6). *, p < 0.05; **, p < 0.01; ***‌, p < 0.001. F-H, mRNA level, lipid peroxidation, MDA and cell counting of enzalutamide-resistant C4-2 and 22Rv1 cell lines after knockdown of FOXA1 (F), CXXC5 (G) or TET2 (H). Data represent mean ± SD (n=3 or 6). ***‌, p < 0.001.

**
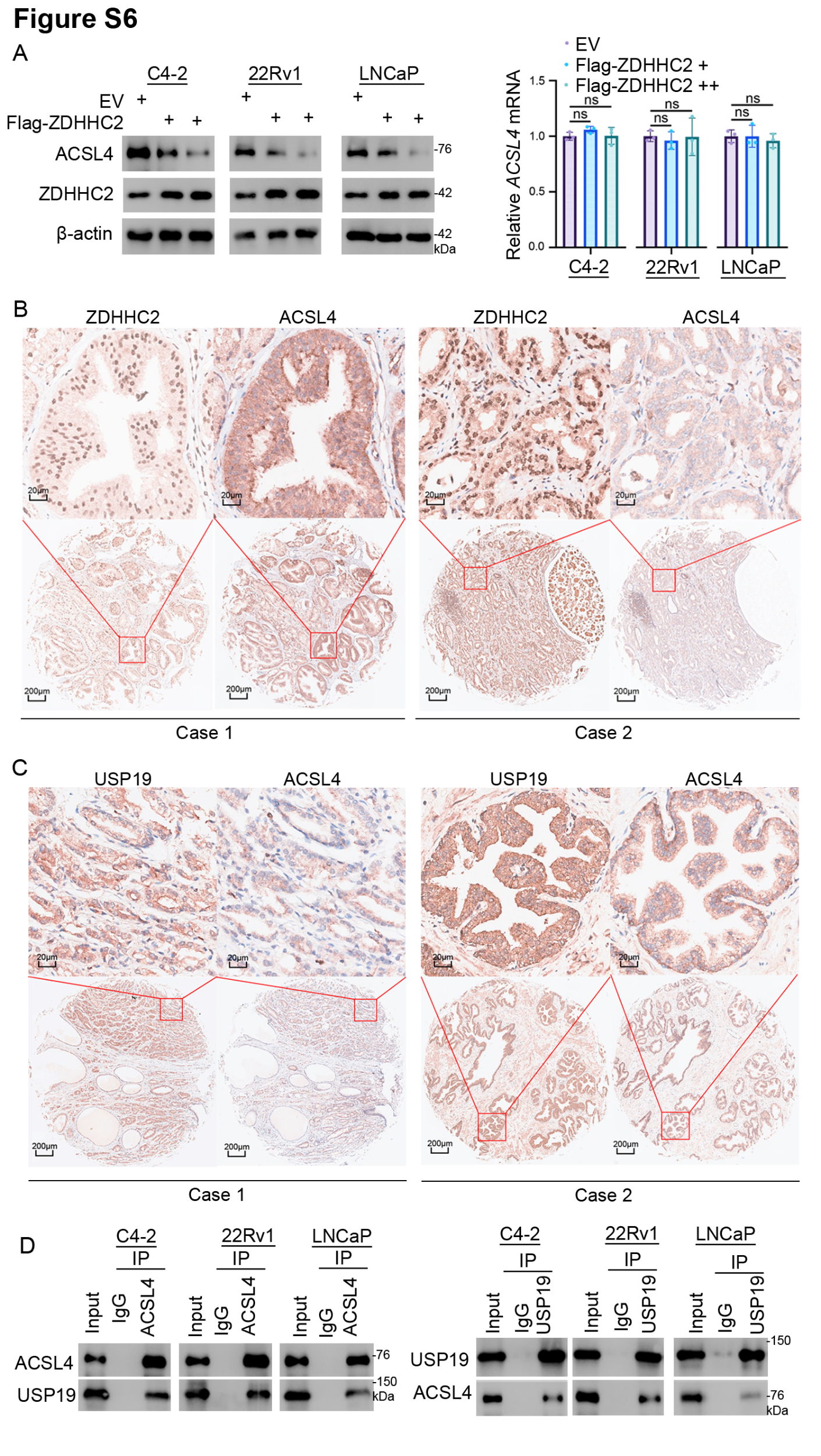
**

**Figure S6. ZDHHC2 promotes the ubiquitin‒proteasome degradation of ACSL4 through USP19**

‌A,‌ C4-2, 22Rv1, and LNCaP cells were transfected with the indicated plasmids for 24 hours. Cells were then collected for Western blot and RT-qPCR analysis. Data represent mean ± SD of three replicates. ns, not significant. ‌B,‌ a prostate cancer tissue microarray was subjected to IHC staining for ZDHHC2 and ACSL4. Representative IHC images are shown. ‌C,‌ a prostate cancer tissue microarray was subjected to IHC staining for USP19 and ACSL4. Representative IHC images are shown. D, C4-2, 22Rv1, and LNCaP cells were harvested for co-immunoprecipitation (co-IP) assays.


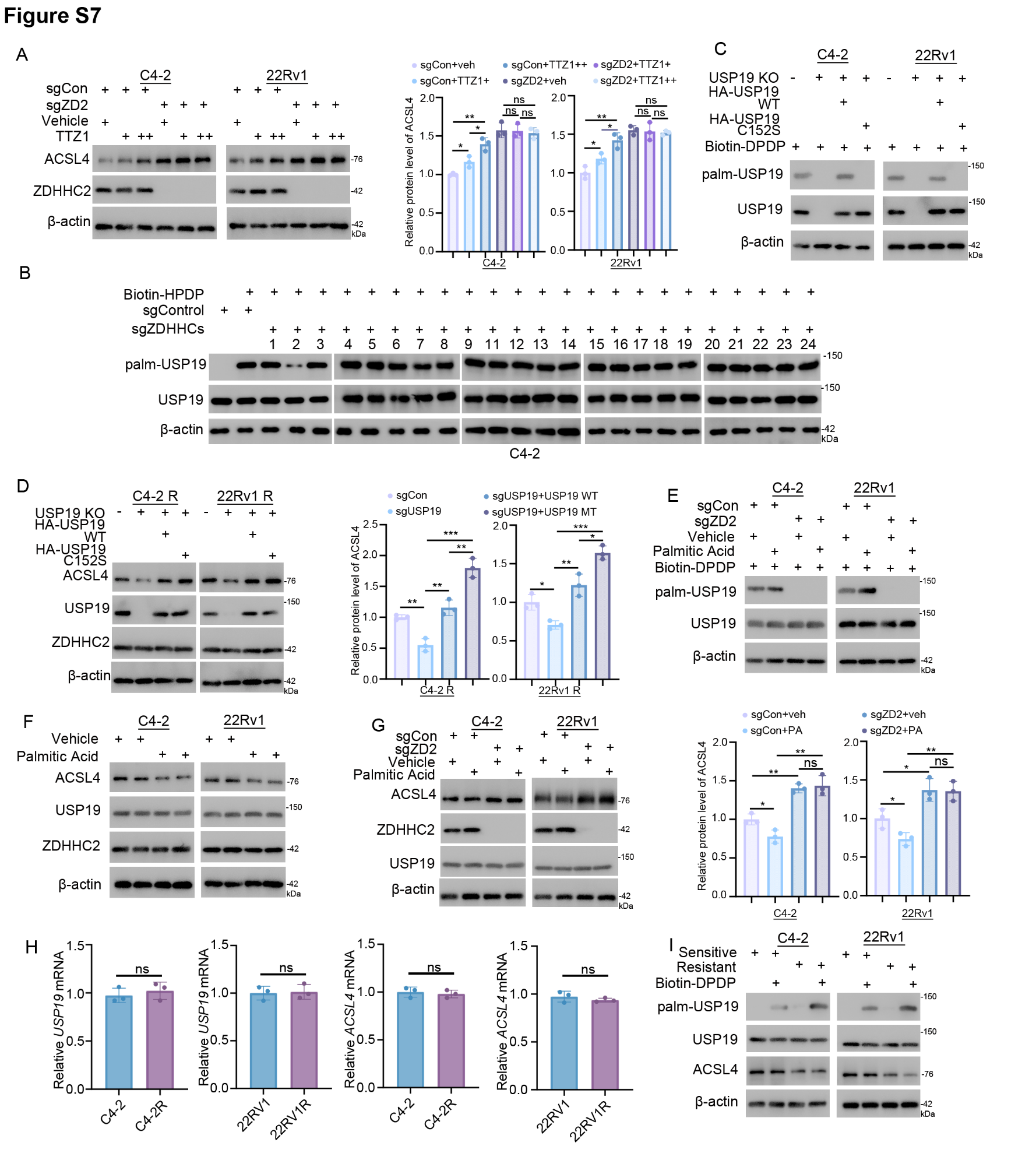


**Figure S7. ZDHHC2 reduces ACSL4 protein level through USP19**

A, endogenous ZDHHC2 was knocked out in C4-2 and 22Rv1 cells with CRISPR/Cas9. The ZDHHC2-knockout and control cells were treated with vehicle or TTZ1 (10 or 20 μM) for 24 hours. And the protein level of ACSL4 was detected by western blot. Repeated for 3 times, and quantitative analysis was carried out. ns, not significant; *, p < 0.05; **, p < 0.01. B, C4-2 cells were infected with lentiviruses containing expression cassettes of control guide RNA or guide RNAs targeting different ZDHHCs and Cas9 protein. ABE assay was used to detect the palmitoylation of USP19. C-D, endogenous USP19 was knocked out in C4-2 and 22Rv1 cells using CRISPR/Cas9. USP19-KO cells were then transfected with indicated plasmids for 24 hours, and detected USP19 palmitoylation level by acyl-biotinyl exchange (ABE) assay (C), and ACSL4 protein level (D). Repeated for 3 times, and quantitative analysis was carried out. *, p < 0.05; **, p < 0.01; ***, p < 0.001. E, endogenous ZDHHC2 was knocked out in C4-2 and 22Rv1 cells using CRISPR/Cas9, and cells were treated with or without palmitic acid for 24 h. The USP19 palmitoylation level was detected by acyl-biotinyl exchange (ABE) assay. F, protein level of ACSL4 was detected after treatment of palmitic acid. G, endogenous ZDHHC2 was knocked out in C4-2 and 22Rv1 cells using CRISPR/Cas9, and cells were treated with or without palmitic acid for 24 h. ACSL4 protein level was detected by western blot. Repeated for 3 times, and quantitative analysis was carried out. ns, not significant; *, p < 0.05; **, p < 0.01. H, mRNA of USP19 and ACSL4 in enzalutamide-sensitive and resistant cell lines. Repeated for 3 times, and quantitative analysis was carried out. ns, not significant. I, The USP19 palmitoylation level was detected by ABE assay in enzalutamide-sensitive and resistant cell lines.

‌


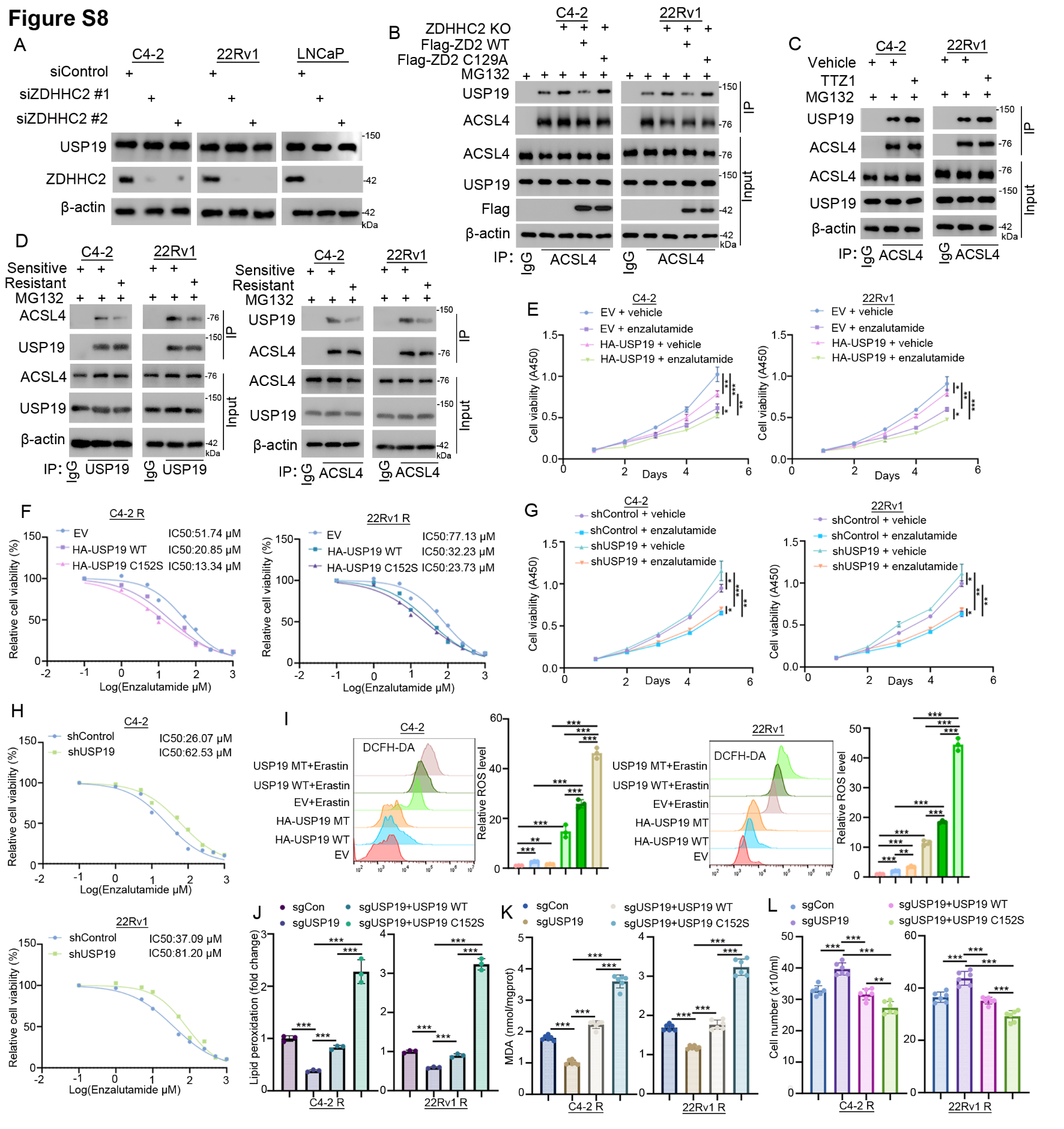


**Figure S8. The palmitoylation of USP19 promotes enzalutamide resistance by inhibiting ferroptosis**

A, C4-2, 22Rv1, and LNCaP cells were transfected with indicated siRNAs for 48 hours. Cells were then collected for Western blot analysis. B, endogenous ZDHHC2 was knocked out in C4-2 and 22Rv1 cells with CRISPR/Cas9. The ZDHHC2-knockout (ZDHHC2-KO) cells were then transfected with indicated plasmids for 24 hours, treated with MG132 and were harvested for co-IP and Western blot analysis. C, C4-2 and 22Rv1 cells were treated with or without TTZ1 and MG132 for 24 hours before collection for co-IP and Western blot analysis. D, co-IP and Western blot analysis were performed in enzalutamide-sensitive and -resistant cell lines. E, C4-2 and 22Rv1 cells were transfected with indicated plasmids for 24 hours. Cells were then treated with or without enzalutamide (10 μM) and subjected to CCK-8 assay. F, enzalutamide resistant C4-2 and 22Rv1 cells were transfected with indicated plasmids for 24 hours, then treated with increasing doses of enzalutamide for 24 hours before CCK-8 viability assays. The corresponding IC50 values are indicated. G, C4-2 and 22Rv1 cells were infected with indicated shRNAs for 72 hours. After puromycin selection, cells were treated with or without enzalutamide (10 μM) and subjected to CCK-8 assay. Data represent mean ± SD of three replicates. **, p < 0.001. H, C4-2 and 22Rv1 cells were infected with indicated shRNAs for 72 hours. After puromycin selection, cells were then treated with a serial dose of enzalutamide for 24 hours and harvested for CCK-8 assay. The corresponding IC50 values are indicated. I, 22Rv1 and C4-2 cells were transfected with indicated plasmids for 24 h, then treated with or without Erastin (10 μM) for 24 h and analyzed for lipid ROS. Data represent mean ± SD of three replicates. **, p < 0.01; ***‌, p < 0.001. J-L, endogenous USP19 was knocked out in enzalutamide-resistant C4-2 and 22Rv1 cells using CRISPR/Cas9. USP19-KO cells were then transfected with indicated plasmids for 24 hours, and analyzed for lipid peroxidation (J), MDA (K) and cell counting (L). Data represent mean ± SD of three or six replicates. Data represent mean ± SD (n=3 or 6). ***, p < 0.001.

**
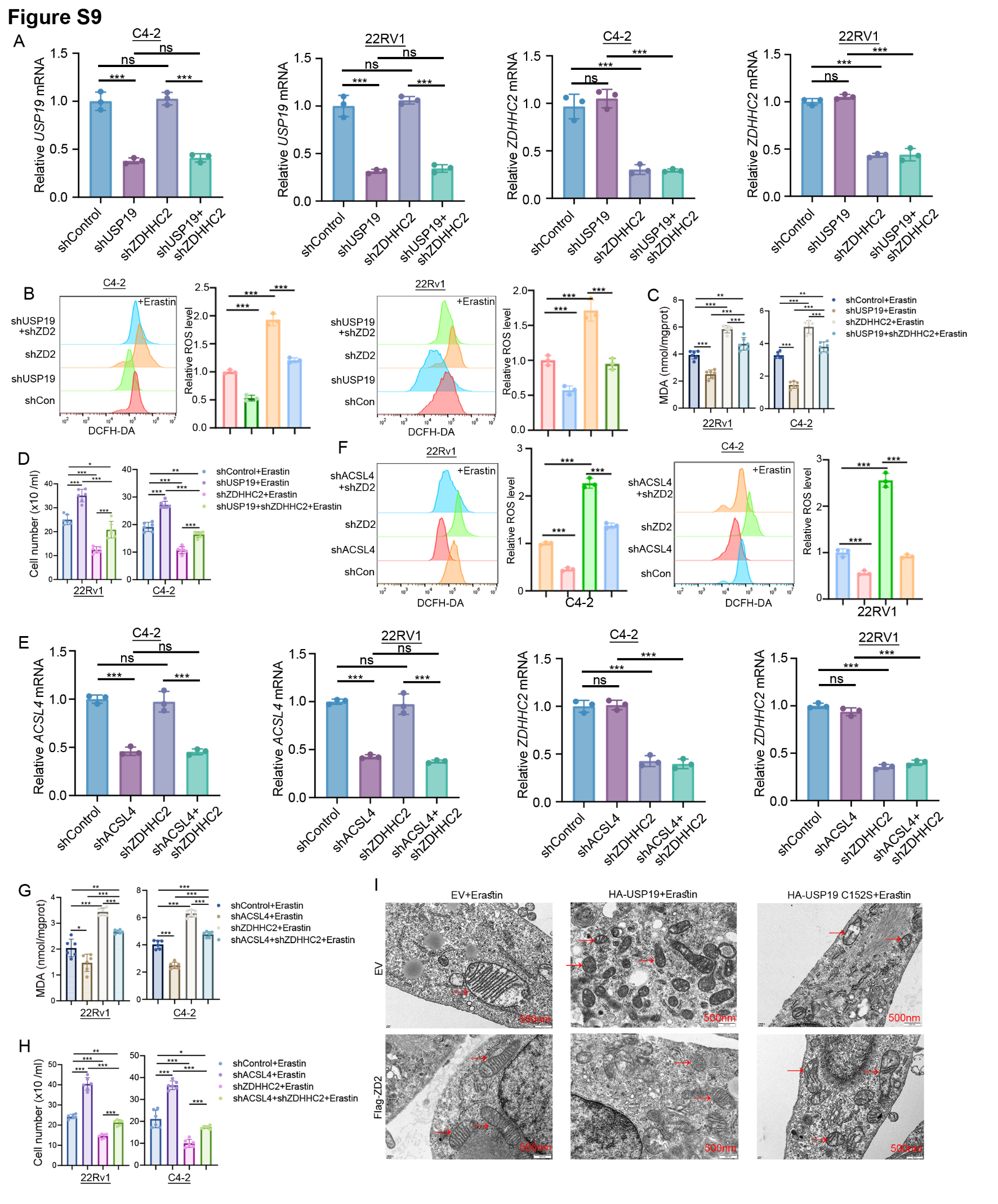
**

**Figure S9 ZDHHC2-USP19-ACSL4 axis inhibits ferroptosis**

A, mRNA level of USP19 and ZDHHC2 was detected. Data represent mean ± SD of three replicates. ns, not significant; ***‌, p < 0.001. B-D, 22Rv1 and C4-2 cells were infected with indicated shRNAs for 72 hours. After puromycin selection, cells were treated with Erastin (10 μM) for 24 hours and subjected to lipid ROS (B), MDA assay (C) and cell counting assay (D). Data represent mean ± SD of three or six replicates. *, p < 0.05; **, p < 0.01; ***, p < 0.001. E, mRNA level of ACSL4 and ZDHHC2 was detected. Data represent mean ± SD of three replicates. ns, not significant; ***‌, p < 0.001. F-H, 22Rv1 and C4-2 cells were infected with indicated shRNAs for 72 hours. After puromycin selection, cells were treated with Erastin (10 μM) for 24 hours and subjected to lipid ROS (F), MDA assay (G) and cell counting assay (H). Data represent mean ± SD of three replicates. *, p < 0.05; **, p < 0.01; ***, p < 0.001. I, 22Rv1 cells were transfected with indicated plasmids for 24 hours. After Erastin (10 μM) treatment for 24 hours, mitochondrial morphology was examined by electron microscopy.

**Table S1. Clinical characteristics of enrolled CRPC patients.**

| **Variable** | **Total (n=10)** | **Enzalutamide-sensitive (n=5)** | **Enzalutamide-resistant (n=5)** | **p value** |
| --- | --- | --- | --- | --- |
| **Age, year** | **71 (64.5-75.5)** | **68 (64-74.5)** | **74 (65-80)** | **0.540** |
| **PSA at diagnosis, ng/mL** | **167.1 (118.1-241.6)** | **198.6 (106.2-247.8)** | **135.6 (111.6-249.5)** | **0.999** |
| **Gleason score** |  |  |  | **0.999** |
| **7** | **3** | **2** | **1** |  |
| **≥8** | **7** | **3** | **4** |  |
| **T stage** |  |  |  | **0.5238** |
| **2** | **1** | **1** | **0** |  |
| **3** | **5** | **3** | **2** |  |
| **4** | **4** | **1** | **3** |  |
| **N stage** |  |  |  | **0.999** |
| **0** | **7** | **4** | **3** |  |
| **1** | **3** | **1** | **2** |  |
| **M stage** |  |  |  | **0.999** |
| **0** | **6** | **3** | **3** |  |
| **1** | **4** | **2** | **2** |  |

**PSA, prostate-specific antigen. Numbers represent median (interquartile range) or N (%).**

**Table S2. The siRNA, shRNA and sgRNA sequences.**

| siZDHHC2 #1 | 5'-GGGCUGAAUUCCACAGCUAAA-3' |
| --- | --- |
| siZDHHC2 #2 | 5'-GGUGAACAAUUGUGUUGGAUU-3' |
| siZDHHC2 #3 | 5'-GGACGGAGAGCAGCAUAAACC-3' |
| siUSP19 #1 | 5'-CGGCUGUUCUUUCCUUCAU-3' |
| siUSP19 #2 | 5'-CAGAGUUGUUGCUCGAUUG-3' |
| siFOXA1 #1 | 5'-GCACUGCAAUACUCGCCUU-3' |
| siFOXA1 #2 | 5'-GGACUUCAAGGCAUACGAA-3' |
| siControl | 5'-UAGCGACUAAACACAUCAA-3' |
| sgZDHHC1 #1 | 5'-GGCGAGCGGAACTACCGGTG-3' |
| sgZDHHC1 #2 | 5'-TGCGGGACAAGAGCTATGCG-3' |
| sgZDHHC2 #1 | 5'-ATGGAAAACACTGGCGAACA-3' |
| sgZDHHC2 #2 | 5'-AGGAGGGTGATGAACACCAC-3' |
| sgZDHHC2 #3 | 5'-GTTGGCATCTGTCACAGTAT-3' |
| sgZDHHC3 #1 | 5'-CCTACACAGTTGTTGACCCA-3' |
| sgZDHHC3 #2 | 5'-GTGGAATCCCACCATGATGA-3' |
| sgZDHHC4 #1 | 5'-AGGATTGGTTCCACAAGTCA-3' |
| sgZDHHC4 #2 | 5'-TCAAGGTCAAGACGTAGATG-3' |
| sgZDHHC5 #1 | 5'-GAACCACGTGAAATCCCGTG-3' |
| sgZDHHC5 #2 | 5'-CAGACCTGAGCCGTTACACA-3' |
| sgZDHHC6 #1 | 5'-AACATATTGCTATAACACCA-3' |
| sgZDHHC6 #2 | 5'-CTTCACTGTGTTCCACCCAA-3' |
| sgZDHHC7 #1 | 5'-AATGGATCATCACTGCCCGT-3' |
| sgZDHHC7 #2 | 5'-AGATGAACTGAAATCCACAA-3' |
| sgZDHHC8 #1 | 5'-ACATCCACGTTCTTGTACAG-3' |
| sgZDHHC8 #2 | 5'-CAGGAACCAAGGATACGTGA-3' |
| sgZDHHC9 #1 | 5'-TGGAAATTCTTGATACGAGG-3' |
| sgZDHHC9 #2 | 5'-AGGCGAAGACATAGATTGTG-3' |
| sgZDHHC11 #1 | 5'-GTGGAAATACATTGCCTACG-3' |
| sgZDHHC11 #2 | 5'-CCAGTACCTCGTGAACCCCG-3' |
| sgZDHHC11B #1 | 5'-GCGATGTATTTCCACGAGTG-3' |
| sgZDHHC11B #2 | 5'-GGAGCACCCTGGGGTTCACG-3' |
| sgZDHHC12 #1 | 5'-GCTGCACATTCACGTAGCCA-3' |
| sgZDHHC12 #2 | 5'-CGTAGCGGCGGACGCAACGG-3' |
| sgZDHHC13 #1 | 5'-GTCATATTATTACTCCAGCA-3' |
| sgZDHHC13 #2 | 5'-TTGTGAGCTGATAACATGAG-3' |
| sgZDHHC14 #1 | 5'-ATACTTACGAAGAATGACGT-3' |
| sgZDHHC14 #2 | 5'-GGTGCCGTTTGCGATATCTG-3' |
| sgZDHHC15 #1 | 5'-TGGAACTTAGAGCGAACACT-3' |
| sgZDHHC15 #2 | 5'-GAATCAAACAGACATACCAA-3' |
| sgZDHHC16 #1 | 5'-CTCAAAGGCAGCATCAACAG-3' |
| sgZDHHC16 #2 | 5'-GTGTGGGCCACTATAACCAT-3' |
| sgZDHHC17 #1 | 5'-GTTGCCAAATATATCCCAAG-3' |
| sgZDHHC17 #2 | 5'-TATTGATGATTACAGCACAT-3' |
| sgZDHHC18 #1 | 5'-CCTGGAGAAACAGATCGGTG-3' |
| sgZDHHC18 #2 | 5'-AGTGTCTGCGACAACTGTGT-3' |
| sgZDHHC19 #1 | 5'-AAGAGGCTAGGGAGGAACCA-3' |
| sgZDHHC19 #2 | 5'-CCTTAACCCGCGCAGCATCG-3' |
| sgZDHHC20 #1 | 5'-GTAGGACCAGACGACCACGA-3' |
| sgZDHHC20 #2 | 5'-AGAACAGATGGAAAGCCACA-3' |
| sgZDHHC21 #1 | 5'-ATCCAGAGTACCATACCTCCATGTGGGATC-3' |
| sgZDHHC21 #2 | 5'-GATCAGTTATGGAGGCCCTCACTAAGGCAA-3' |
| sgZDHHC22 #1 | 5'-AGAACTCCCCAGACGACCTG-3' |
| sgZDHHC22 #2 | 5'-GGAATAGGAAGAGCGCCCCG-3' |
| sgZDHHC23 #1 | 5'-CGAGTACATAGATCGGAATG-3' |
| sgZDHHC23 #2 | 5'-TACCTCAGCAATCCAGCAAG-3' |
| sgZDHHC24 #1 | 5'-CCTGTGAGCAACATGAGCCA-3' |
| sgZDHHC24 #2 | 5'-GGCCGGCCAGCATCACGCCA-3' |
| sgFOXA1 #1 | 5'-GGTTCATGGCGGCCGCGTAGGGG-3' |
| sgFOXA1 #2 | 5'-TTGGTAGTACGCCGGCTCCAGGG-3' |
| sgUSP19 #1 | 5'-AAGATACCTCCGCCAAACGCAGG-3' |
| sgUSP19 #2 | 5'-AGGCATAACGCGTCACAACCTGG-3' |
| shControl | 5'-CCGGCAACAAGATGAAGAGCACCAACTCGAGTTGGTGCTCTTCATCTTGTTGTTTTT-3' |
| shZDHHC2 #1 | 5'-CCGGGCTCCGTCTGTGATAAATGTACTCGAGTACATTTATCACAGACGGAGCTTTTTG-3' |
| shZDHHC2 #2 | 5'-CCGGGCCAAGGATCTTCCCATCTATCTCGAGATAGATGGGAAGATCCTTGGCTTTTTG-3' |
| shAR #1 | 5'-CCGGGTAATAGTGGTTACCATTCATCTCGAGATGAATGGTAACCACTATTACTTTTTG-3' |
| shAR #2 | 5'-CCGGGTAGTTGTGAGTATCATGATTCTCGAGAATCATGATACTCACAACTACTTTTTG-3' |
| shCXXC5 #1 | 5'-CCGGCAACAGAAGAAAGGGCTTCTTCTCGAGAAGAAGCCCTTTCTTCTGTTGTTTTTTG-3' |
| shCXXC5 #2 | 5'-CCGGGAAAGACTGGCCATCAGATTTCTCGAGAAATCTGATGGCCAGTCTTTCTTTTTTG-3' |
| shTET2 #1 | 5'-CCGGAGTGTTCCGCAATTTACATCTCGAGATGTAAATTGCGGAACACTTTTTTG-3' |
| shTET2 #2 | 5'-CCGGGTTTATCCAGAATTAGCAACTCGAGTTGCTAATTCTGGATAAACTTTTTG-3' |
| shTRIM33 #1 | 5'-CCGGTACTTTCCAGTTGCGTCATATCTCGAGATATGACGCAACTGGAAAGTATTTTTG-3' |
| shTRIM33 #2 | 5'-CCGGGATGCTGGCTCAAGTAGTTTACTCGAGTAAACTACTTGAGCCAGCATCTTTTTG-3' |
| shFBXW5 #1 | 5'-CCGGCGTGCGGAACAAGCCCTATGACTCGAGTCATAGGGCTTGTTCCGCACGTTTTTG-3' |
| shFBXW5 #2 | 5'-CCGGCGACGAGTGCTTCTTCATCTTCTCGAGAAGATGAAGAAGCACTCGTCGTTTTTG-3' |
| shUSP36 #1 | 5'-CCGGACAGAACATCCAACGTCTTAACTCGAGTTAAGACGTTGGATGTTCTGTTTTTTG-3' |
| shUSP36 #2 | 5'-CCGGCTACCTTGGTCCATCAAATTTCTCGAGAAATTTGATGGACCAAGGTAGTTTTTG-3' |
| shUSP19 #1 | 5'-CCGGCGGCACAAGATGAGAAATGATCTCGAGATCATTTCTCATCTTGTGCCGTTTTTG-3' |
| shUSP19 #2 | 5'-CCGGCGATCCTTTGAAGCTGAGATTCTCGAGAATCTCAGCTTCAAAGGATCGTTTTTG-3' |
| shACSL4 #1 | 5'-CCGGGCAGTAGTTCATGGGCTAAATCTCGAGATTTAGCCCATGAACTACTGCTTTTTG-3' |
| shACSL4 #2 | 5'-CCGGCCAGTGTTGAACTTCTGGAAACTCGAGTTTCCAGAAGTTCAACACTGGTTTTTG-3' |
| shFOXA1 #1 | 5'-CCGGGCGTACTACCAAGGTGTGTATCTCGAGATACACACCTTGGTAGTACGCTTTTTG-3' |
| shFOXA1 #2 | 5'-CCGGGAACTCCATGAACACCTACATCTCGAGATGTAGGTGTTCATGGAGTTCTTTTTG-3' |

**Table S3. The primer sequence for RT-qPCR.**

| Gene（Human） | Forward primer (5′ - 3′) | Reverse primer (5′ - 3′) |
| --- | --- | --- |
| β-actin | 5'-GCGTGACATTAAGGAGAAG-3' | 5'-GAAGGAAGGCTGGAAGAG-3' |
| ZDHHC2 | 5'-CTTGCTGGTTGGCGTCAATG-3' | 5'-AGGAGCAGCCATCACCTAGA-3' |
| AR | 5'-GGTGAGCAGAGTGCCCTATC-3' | 5'-GAAGACCTTGCAGCTTCCAC-3' |
| CXXC5 | 5'-GTTTGCGCAGTCCACAGAGA-3' | 5'-CTCTCCCTGCATGGGGTACT-3' |
| TET2 | 5'-AGGCTAGGCTGCTTTCGTAG-3' | 5'-GAATGTTTGCCAGCCTCGTT-3' |
| ID1 | 5'-GCTCTACGACATGAACGGCT-3' | 5'-GGGGTTCCAACTTCGGATTC-3' |
| PFN2 | 5'-TGGCAGAGCTACGTGGATAAC-3' | 5'-AAACCTTCCCGGTCTTTTCCT-3' |
| KLK3 | 5'-GTCTGCGGCGGTGTTCTG-3' | 5'-TGCCGACCCAGCAAGATC-3' |
| ACSL4 | 5'-TGTGGACAATAAGGCTATCA-3' | 5'-TGGTCTACTTGGAGGAATG-3' |

**Table S4. The primer sequence for ChIP-qPCR.**

| Gene（Human） | Forward primer (5′ - 3′) | Reverse primer (5′ - 3′) |
| --- | --- | --- |
| ZDHHC2 | 5'-GGAGCGGGTTGAGGAAAAGA-3' | 5'-GGGAAGCAGAAATGCACAGC-3' |

**Table S5. List of significantly changed ferroptosis-related genes in proteomics of clinical samples.**

| Gene Symbol | P.value | FC | Log2FC | FDR | Significant |
| --- | --- | --- | --- | --- | --- |
| GCLC | 0.028288 | 2.384015 | 1.253393 | 0.757031 | Up |
| NFS1 | 0.029936 | 1.675611 | 0.744688 | 0.757031 | Up |
| CBS | 0.032379 | 2.439599 | 1.286644 | 0.757031 | Up |

**Table S6. List of significantly changed ferroptosis-related genes in palmitoyl-proteomics of clinical samples.**

| Gene Symbol | P.value | FC | Log2FC | FDR | Significant |
| --- | --- | --- | --- | --- | --- |
| GPX4 | 0.004255 | 2.494359 | 1.318669 | 0.015879 | Up |
| SLC1A5 | 0.004113 | 2.90607 | 1.53907 | 0.015489 | Up |
| ACO1 | 0.048066 | 0.393625 | -1.34511 | 0.114998 | Down |

**Table S7. List of significantly changed ferroptosis-related genes in proteomics of cell lines.**

| Gene Symbol | P.value | FC | Log2FC | FDR | Significant |
| --- | --- | --- | --- | --- | --- |
| **ACSL4** | 2.64E-05 | 0.598327 | -0.74099 | 0.010763 | Down |
| FADS2 | 0.000342 | 0.411988 | -1.27933 | 0.014985 | Down |
| ACSF2 | 0.037039 | 0.593524 | -0.75262 | 0.107365 | Down |
| GPX4 | 0.002745 | 1.548083 | 0.630483 | 0.027086 | Up |
| FTH1 | 0.006849 | 1.5677 | 0.648649 | 0.042622 | Up |
| CHAC1 | 0.006855 | 1.741823 | 0.800598 | 0.042622 | Up |

**Table S8. List of significantly changed ferroptosis-related genes in palmitoyl-proteomics of cell lines.**

| Gene Symbol | P.value | FC | Log2FC | FDR | Significant |
| --- | --- | --- | --- | --- | --- |
| ABCC1 | 0.021408 | 0.417973 | -1.25852 | 0.086442 | Down |
| ACACA | 0.001355 | 2.109357 | 1.076803 | 0.018488 | Up |
| ACO1 | 0.003338 | 0.498648 | -1.00391 | 0.028523 | Down |
| ACSF2 | 0.000522 | 4.871382 | 2.284331 | 0.012758 | Up |
| C10orf70 | 9.72E-05 | 0.030013 | -5.05826 | 0.00601 | Down |
| CARS | 0.004518 | 2.456601 | 1.296664 | 0.033451 | Up |
| CD44 | 0.004698 | 6.518245 | 2.704484 | 0.033976 | Up |
| FANCD2 | 0.007708 | 2.258639 | 1.175454 | 0.04588 | Up |
| FDFT1 | 9.44E-05 | 0.291134 | -1.78024 | 0.005962 | Down |
| GCLC | 0.003563 | 2.09827 | 1.0692 | 0.029491 | Up |
| GSS | 0.041691 | 2.02929 | 1.020975 | 0.132671 | Up |
| KEAP1 | 0.004057 | 2.269712 | 1.182509 | 0.031512 | Up |
| NCOA4 | 7.27E-05 | 0.140179 | -2.83466 | 0.005351 | Down |
| PHKG2 | 0.001416 | 0.389366 | -1.3608 | 0.018842 | Down |
| RPL8 | 0.001015 | 5.51047 | 2.462175 | 0.015825 | Up |
| STEAP3 | 0.001907 | 0.091447 | -3.45092 | 0.02091 | Down |
| TFRC | 0.004082 | 5.61977 | 2.490511 | 0.031538 | Up |
